# Partial purification and characterization of protease extracted from *kinema*

**DOI:** 10.1016/j.heliyon.2024.e27173

**Published:** 2024-02-27

**Authors:** Dambar Bahadur Khadka, Tikaram Pahadi, Sunil Aryal, Dhan Bahadur Karki

**Affiliations:** a Central Department of Food Technology, Tribhuvan University, Dharan, Nepal; b Central Campus of Technology, Tribhuvan University, Dharan, Nepal

**Keywords:** *Kinema*, Protease, Enzyme extraction, Partial purification, Characterization

## Abstract

Proteases are large group of highly demanded enzymes having huge application in food and pharmaceutical industries. Numerous sources, including plants, microorganisms, and animals, can be used to obtain protease. Due to its affordability and safety consideration, fermented foods have recently attracted more attention as a source of microbial protease. The present study aimed to extract protease from *kinema*, partially purify the extracted protease following dialysis after precipitation with ammonium sulfate, and determine general characteristics of protease. The *kinema* having highest proteolysis activity after three days of control fermentation (Temperature 30±2 °C, RH 66 ± 2%) was taken for the study. About 2.45 fold of purification with overall recovery of 63.21% was achieved after precipitation with ammonium sulfate at 30–70% saturation level followed by dialysis of crude extracted protease. The dialysed *kinema* protease had specific activity of 7.90 U/mg. The enzyme remained actively functional across a wider pH (5–9) and temperature (40-60 °C) range. SDS-PAGE and Zymogram confirmed the presence of three major active bands respectively of 29.04 kDa, 36.09 kDa and 46.35 kDa in the kinema protease extract. The enzyme kinetics data on casein, fitted to Mechaelis Mentens’ plots showed the protease had V_max_ of 1.001 U/ml with corresponding K_m_ value of 0.825 mg/ml. Metal ions such as iron, mercury and aluminium showed the inhibition effect whereas presence of sodium, zinc, and calcium shows the activation effect on protease performance. The enzyme was active over various natural substrates; showing maximal activity on casein, and subsequent to bovine serum albumin, gelatin, hemoglobin and whey protein respectively. Furthermore, molecular weight distribution of the protease extract and activity inhibition with ethylenediaminetetraacetic acid and phenylmethylsulfonyl fluoride, suggesting the protease from *kinema* could be a metal dependent serine protease or mixture of them.

## Graphical abstract

**Figure:**
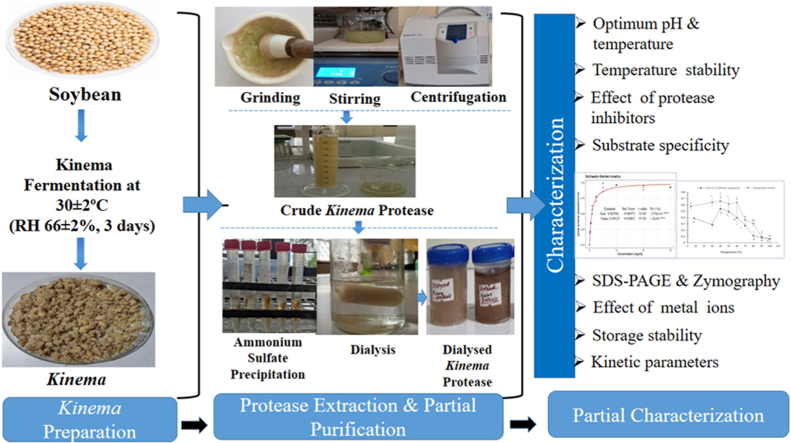

## 1 Introduction

After carbohydrases, proteases represent the second biggest class of commercial hydrolytic enzymes, and demand is rising globally [1]. They are used in numerous industrial processes, including those in the pharmaceutical, food, textile, hide and feed [2,3]. Due to the lack of sufficient animal production and other issues such as ethical, cultural and religious; interest toward alternative sources (microbial and plant sources) is increasing [4,5]. Papain from *papaya latex* and fruit, bromelain from pineapple peel, fruit, crown, and actinidin from kiwi fruit have been already commercialized plant cysteine protease. Whereas cysteine proteases such as ficin from fig, zingibain from ginger and serine proteases such as cucumisin (cucumisin like) from cucurbits, have been reported as the new emerging plant proteases [6]. Similarly, various microbial proteases have been commercialized, among them proteases from *Bacillus* sp. *(*Neutrase, thermolysin and alcalase) and *Aspergillus* sp. (Flavourzyme, Corolase etc.) are extensively studied and exploited [7].

Microbial proteases enable fast production, have less influences of climatic or seasonal changes [8]. Bacteria are among the most significant groups of microorganism that produce proteases. Even among bacteria, the species *Bacillus* is regarded as one of the most prominent sources of protease because of its capacity to release substantial quantity of protein, produces both alkaline and neutral extracellular protease [2]. They can be used to produce protease by submerged as well as solid state fermentation, and can be manipulated genetically to enhance the production [[8], [9], [10]]. Though they are abundant in natural sources, interest of researcher are increasing toward the fermented food as an alternative sources of the proteases and proteolytic strains, as they are generally recognized as safe [[8], [9], [10]].

Recent studies have shown that soybean-based traditional fermented food such as Japanese *natto* [11,12], *thua-nao* from northern Thailand [13], *gembus* from Indonesia [14], Korean *doenjang* [15], *cheonggukjang* [16] as a potential alternative sources of proteases. The protease having homology with subtilisin also known as nattokinase (Subtilisin NAT) has been already extracted, purified and characterized from Japanese *natto* and has shown broad specificity toward various synthetic substrates [17,18]. Nattokinase is one of the most studied and commercial fibrinolytic proteases from soybean-fermented food; mainly used in therapeutic applications for the treatment of thrombotic disorder and thrombovascular diseases [19]. The potential applications of nattokinase as an additives in functional foods and in dairy processing are also emerging [12,19]. Additionally, various strains (mainly of *Bacillus* sp.) have also been isolated, and applied as sources of protease from soybean-fermented foods such as *thua-nao* [13]*, natto* [11,12,20]*,* and *douchi* [21].

Over the years, many fibrinolytic enzymes were purified and characterized from Asian fermented food; they have been reported to be either serine proteases such as nattokinase from *natto* [17], Subtilisin DFE from *douchi* [22], CK from *chungkook-jang* [23], APR68 from *meju* [24] or metalloprotease such as NPR68 from *meju* [24]; MCE [12], B_12_ nattokinase [11] from *nattto*. Fibrinolytic serine protease from fermented food are mentioned to be neutral to alkaline; have optimum pH between 8 and 10 and optimum temperature 30-70 °C. While metalloprotease have optimal pH between 6 and 7 and optimum temperature between 33 and 50 °C [9].

*Kinema* is a non salted, alkaline soybean fermented food with an ammonical pungent smell and a slimy texture [25]. It is traditionally prepared and consumed by the non-Brahmin communities living in eastern hilly districts of Nepal, Sikkim and Darjeeling of India, and some areas of Bhutan [26]. It is produced by solid-state fermentation process, and *Bacillus subtilis* is reported as the major dominating organism involved in fermentation [27]. An increase in soluble nitrogen during *kinema* fermentation indicated extensive proteolysis activity and involvement of proteases [28,29]. Recently, a thermally resistant osmotolerant *B. amyloliquefaciens* BKHE strain was isolated from *kinema* and tested for the ability to produce alkaline *protease* [30]. These facts support that *kinema* possess tremendous opportunity as a source of protease or proteinase enzymes. In spite, kinema compared to *natto* and *thua-nao*, has been less studied so far as the source of proteases and proteolytic strains, and also for purification and characterization of proteases. *Kinema* could be a valuable novel and cheap source of protease or proteolytic strains, and it could be one alternative to valorize *kinema* by evaluating the proteolytic activity and characterizing the proteolytic extract in terms of molecular weight distributions, types of proteases, substrate specificities and other general characteristics. Thereby, this study was purposed to evaluate general characteristics of *kinema* protease extract after partial purification. Partial purification was accomplished by dialysis of selective ammonium sulfate precipitates obtained from the crude extract. The information obtained from the study could also be essential for further purification of *kinema* proteases and to evaluate their biotechnological and industrial applications in the future.

## 2 Materials and methods

### 2.1 Collection of soybean

The yellow variety of the soybean (*Glycine max (L.)* Merrill) was procured from the local market of Dharan, Nepal. The collected grains were cleaned and sorted manually to remove dust, foreign matter and damaged ones.

### 2.2 Preparation of *kinema*

The cleaned and sorted soybean seeds were subjected to washing with clean water to get rid of remaining dirt, dust and mud and then used for *kinema* preparation with some modifications [25]. Soybean was soaked overnight (in water with a regular exchange of water). Soaked soybean seeds were cooked in water for 2 h in a well-covered vessel till the beans became soft. After draining of excess water, the cooked seeds were gently cracked by splitting the cotyledon using a mortar and pestle. The split seeds were mixed thoroughly with 1% firewood ash, kept in a bamboo container coated inside by banana leaves and wrapped. It was further covered by muslin cloth and kept for spontaneous fermentation at 30 ± 2 °C in an environmental/stability cabinet chamber (Navayuga, India) for 72h. The microorganism present in the soybean, as well as in the machinery, firewood ash, and wrapping materials, provides a source for the fermentation of *kinema* [27].

### 2.3 Determination of proteolysis during *kinema* fermentation

*Kinema* sample was taken at every interval of 24h during the fermentation period of 0–6 days. Samples (3g) were homogenized in 4 ml of phosphate buffer (pH7.4, 50 mM) on a magnetic stirrer for 20min at refrigerated condition. The homogenate was filtered through the clean muslin cloth, and centrifuged (Refrigerated centrifuge, Sigma 3-30 KS) at −4 °C and 7000 rpm for 10 min. The supernatant was collected after filtration (using whatman 41 filter paper), volume maintained at 4 ml with the same buffer in a clean test tube. The extract’s protein content and protease activity were assessed respectively by Bradford [31] and Cupp-Enyard [32] methods.

### 2.4 Proximate analysis of soybean and *kinema*

Soybean and the *kinema* after completion of three days of fermentation were analyzed for proximate composition. The hot air oven drying method at 130 ± 2 °C (until to get constant weight) was applied for estimating the moisture content [33]. Crude protein was indirectly derived from the total amount of nitrogen obtained from micro Kjeldahl method using converting factor of 5.7 [33]. The amount of ash was measured by igniting the sample in the muffle furnace at 600 °C until the difference between two subsequent weighing was less than 1 mg [33]. The solvent (petroleum ether) extraction method was used to determine crude fat [34]. Carbohydrate content was determined by difference method.

### 2.5 Extraction and partial purification of crude protease

Protease enzyme was extracted from three days fermented *kinema* with the solvent sodium phosphate buffer (pH 7.4, 50 mM) keeping *kinema*: solvent ratio of 1: 1.5 (w/v). For this, 80g of *kinema* added to 120 ml buffer, stirred by magnetic stirrer at 150 rpm for 20 min. After stirring, the whole content was filter through the clean muslin cloth and volume made up to 120 ml followed by centrifugation (Sigma 3-30 KS at −4 °C) for 12 min at 13000 rpm. The supernatant obtained after centrifugation and filtration through the muslin cloth was collected as crude *kinema* protease [35].

The study aimed to valorize *kinema* as a potential source of protease and to observe diversity of major proteases distributed in the *kinema* extract. As a result, only one step of purification using ammonium sulfate precipitation and dialysis was performed. Partial purification of the crude *kinema* protease was obtained by the ammonium sulfate precipitation followed by dialysis as described by Abd-Eikhalek et al. [36]. Firstly, the crude protease (10 ml) was fractionated by increasing concentration of ammonium sulfate from 0 to 20%, 20–30%, 30–40%, 40–60% 60–70% and 70–80% saturation level followed by centrifugation at refrigerated centrifuge (13000 rpm for 12 min, at −4 °C) to determine the salt cut off level [37]. The pellet from each fraction was dissolved in 2 ml of sodium phosphate buffer (pH 7.4, 50 mM) and subjected to assay for protease activity and determination of protein.

After determining salt cut off level, the remaining crude enzyme (100 ml) was precipitated again at that cut off level of ammonium sulfate saturation, and was dialyzed to remove excess ammonium sulfate concentration as mentioned by Sachin et al. [38]. Dialysis-tube (Molecular weight cut off 12 kDa, Himedia LA395) was pretreated according to manufacturer protocol to remove sulfur compounds and additives. The crude *kinema* protease was subjected to dialyze against four exchanges of phosphate buffer (pH 7.4, 50 mM) in the refrigerated condition to obtain a partially purified *kinema* protease. At every steps of purification, samples were analyzed for protease activity and protein content.

### 2.6 Determination of protein content

The protein presence in crude and partially purified protease were quantified by employing the method of Bradford [31]. Briefly, 0.1 ml of the enzyme was combined with 3 ml of Bradford reagent, allowed to develop color for 30 min at 37 °C, and then absorbance was observed at 595 nm in a spectrophotometer (Carry 60 UV–Vis, Agilent, USA). Protein quantification was done by comparing with a standard curve of BSA (0–1000 μg/ml) and presented in milligrams per milliliter (mg/ml).

### 2.7 Determination of protease activity

Protease activity was evaluated using Cupp-Enyard [32] method with some modification. 100 μl of crude enzyme was added to 2.2 ml casein (5 mg/ml) solution prepared in 50 mM sodium phosphate buffer having 7.4 pH. Following 10 min of incubation at 40 °C, 3.5 ml of 5% Trichloroacetic acid (TCA) was added to stop the reaction. For the preparation of blank, enzymes was added only after addition of TCA to the casein. The reaction mix was refrigerated for 15 min, centrifuged at 7000 rpm for 7 min. After then, TCA filtrate was collected after filtration with Whatmann No. 41 filter paper. One milliliter of TCA filtrate was added to 2% sodium carbonate (Na_2_CO_3_) reagent. After standing for about 10 min at room temperature, 0.5 ml diluted folin-ciocalteu reagent ((1:1) was added. The reaction mix was incubated for 30 min at 37 °C, and absorbance was taken at 700 nm by UV–Vis Spectrophotometer (Carry 60 UV–Vis, Agilent). As a standard, tyrosine (0–100 μg/ml) was used to create a standard curve.

$$\text{Protease}\;\text{activity}\;\left(U/\text{ml}\right)=\frac{\mu \text{mole}\;\text{tyrosine}\;\text{equivalent}\;\text{released}\times \text{Vt}}{\text{Ve}\times t\times \text{Vc}\;}$$

Where, V_t_; total assay volume (ml), V_e_; enzyme volume (ml), t; hydrolysis time (min) and V_c_;TCA filtrate volume (ml).

Specific activity (U/mg) was calculated by dividing the protease activity (U/ml) by protein content of the enzyme (mg/ml). ‘The amount of enzyme that released 1 μmole tyrosine per ml in 1 min under the given assay conditions was considered as one unit of protease activity’ [32].

### 2.8 General characterization of protease

#### 2.8.1 Determination of optimum pH

*kinema* protease activity at 40 °C was investigated applying pH ranges of 4–10; to determine optimal pH [39]. Carbonic acid buffer (10 pH), Tris-HCl buffer (8 and 9 pHs), sodium phosphate buffer (6 and 7 pH) and citrate buffer (4 and 5 pHs) were used to achieve this. Casein solution (5 mg/ml) was prepared in each buffer and subjected to determine protease activity as described earlier.

#### 2.8.2 Determination of optimum temperature and temperature stability

To determine influence of temperature, enzymatic activity was assayed in the temperature ranges of 30–100 °C on casein [39]. All the reactions were performed at pH 7.4; in each of test temperatures maintained in the water bath. The enzyme and casein solution were kept at each tested temperature for 5 min prior to mixing and conducting the reactions.

The protease stability against the temperatures was determined as per the procedure outlined by Tomar et al. [40] with some modifications. Protease solution was first exposed to various temperatures (5 °C, 28 °C, 50 °C, 60 °C, 70 °C, 80 °C, 90 °C and 100 °C) for 30 min. An aliquot was then used for hydrolysis reactions to analyze remaining protease activity.

#### 2.8.3 Determination of substrate specificity

Partially purified enzyme’s activity was analyzed in presence of substrates; casein, BSA, whey protein, hemoglobin and gelatin, with some modification [39]. Protease assay was performed as described earlier at 5 mg/ml concentrations of each substrate, prepared in sodium phosphate buffer (50 mM, pH 7.4) and the result was expressed in term of relative activity considering activity on casein as 100%.

#### 2.8.4 Determination of kinetic parameters

The effect of increased casein concentration ranges from 0 to 30 mg/ml on enzyme kinetics was studied as per the method described by Tomar et al. [40]. Protease assay was used to perform measurements at optimized pH and temperature as described earlier. TCA was added prior to addition of enzyme to the casein for each of the concentrations for preparation of the blanks. The kinetic data were fitted into Mechaelis-Mentens curve and then to Lineweaver-Burk plot by using R 4.3.0 (Package drc and ggplot_2_) to calculate kinetic parameters; maximum velocity of reaction (V_max_) and Mechaelis Mentens’ constant (K_m_).

#### 2.8.5 Inhibition or activation effect of metal ions

Assay for *kinema* protease activity was conducted after adding monovalent (Na^+^, K^+^), divalent (Ca^++^, Zn^++^, Cu^++^, Hg^++^, Co^++^) and trivalent (Al^+++^, Fe^+++^) metal ions at 5 mM concentration in a reaction mixture to study their effects [39]. The percentage of relative activity compared to the control (no additional metal ions) activity was used to express the results.

#### 2.8.6 Inhibition by protease inhibitors

Residual activity of partially purified *kinema* protease after pre-incubation with of 5 mM protease inhibitors; phenylmethylsulfonyl fluoride (PMSF, a serine inhibitor), Idoacetamide (IDA, a cysteine inhibitor), and ethylenediaminetetraacetic acid (EDTA, a metalloprotease inhibitors) were conducted [39]. The 200 μl enzyme was first treated with 200 μl of 10 mM concentration of each of the inhibitors for 1h at 37 °C. After then, residual activities were assessed by performing protease assay, and reported as percentage of residual activity relative to control (absence of inhibitors) activity.

#### 2.8.7 Determination of storage stability

The stability of the enzyme was tested for 16 days of storage at both at 4 °C (Refrigerated condition) and −20 °C (Deep freeze condition) according to the method described Gagaoua et al. [41]. The assay was done for protease activity at every two days interval; and the result are expressed in term of % of the activity of the enzyme at zero day.

#### 2.8.8 Electrophoretic analysis and zymography

Using Tricine SDS–PAGE in accordance with the methodology of Laemmli [42], the pattern of *kinema* proteases were assessed. The sample buffer (1.25 M Tris–HCl, pH 6.8, 4% SDS, 20% glycerol) and protease extract were combined in a 1:1 ratio, heated in water bath at 85°Cfor 10 min. Ten microgram of sample was loaded onto a gel (composed of 4% stacking and a 15% separation gel). At room temperature, electrophoresis was carried out constantly at voltage of 100V. Following electrophoresis, staining of gel was done for entire night using solution comprised of 0.1% Coomassie Brilliant Blue (CBBR -250), 10% acetic acid and 45% methanol. Finally destaining was performed two times using destaining solution containing 7.5% acetic acid 50% methanol. A prestained protein ladder with range 10–250 kDa (Thermo scientific) was used as standard. The graph of log molecular weight versus relative mobility of markers was utilized to estimate molecular wieight of the enzyme.

In order to confirm protease activity and most active bands present in *kinema* protease extract, gelatin zymography was conducted using 4% stacking gel and 8% separating [40]. 0.1% gelatin was incorporated during separating gel preparation to facilitate zymography. Samples were loaded to the gel at concentration of 5 μg, 7 μg and 10 μg after being combined in 1:1 ratio with non-reducing sample buffer. Electrophoresis was run at a fixed 100V voltage. Successively, the gel was immersed in renaturing buffer consist of 2.5% Triton X-100 for 1 h, then incubated for whole night at 37 °C in 50 mM (pH8) Tris buffer containing 0.2 M NaCl and 5 mM Cacl_2_. The gel was finally stained using 0.2% CBBR-250 for 30 min. Protease activity was identified as a bright zone appeared on the destained gel.

### 2.9 Data analysis

All the test was conducted in triplicate. Results were expressed in average value ± SD. The experimental data were calculated and the graphically interpreted by using Microsoft Excel 2016. IBM SPSS version 20 was used for the statistical analysis. Analysis of variance (ANOVA) was applied to find significance difference among the sample mean values, and Tukey’s HSD test for multiple comparison at 5% level of significance.

## 3 Results and discussion

### 3.1 Proximate composition of soybean and *kinema*

The proximate composition of the soybean and the prepared *kinema* (after three days of fermentation) are presented in Table 1. The results obtained were within the ranges reported by the various researchers [25,26,28,43].

**Table 1:** Proximate composition of soybean and *kinema*.

Parameters^a^	Soybean	*kinema*
Moisture (%)	8.89	63.40
Crude protein (% db)	40.20	45.00
Crude fat (% db)	20.42	23.50
Total ash(% db)	4.43	6.10
Crude fiber (% db)	4.55	3.20
Carbohydrate (% db)	30.40	22.20

a db: Dry basis.

### 3.2 Proteolysis during *kinema* fermentation

**Fig. 1:**
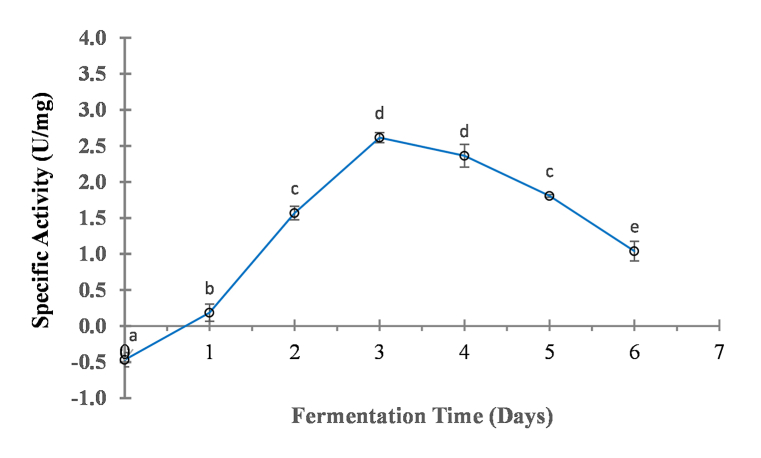
Specific activity of crude protease extracted from *kinema* at different fermentation time Value are mean of triplicate ± SD, Mean value with different alphabet are significantly different at 5% level of significance (p < 0.05). *Kinema* sample were drawn aseptically at every day from *kinema* fermentation mass. Specific activity was determined by dividing protease activity (U/ml) by protein content of the *kinema* extract; 3g sample was homogenized with 4 ml sodium phosphate buffer (pH7.4, 50 mM) to prepare the extract).

The protein content and protease activity of *kinema* sample from different fermentation days (0–6 days) were examined and the result were expressed in term of specific activity (Fig. 1.). Higher specific activity 2.62 ± 0.007 U/mg protein (Protease activity; 0.208 U/ml) and protein content 0.068 mg/ml) was observed on 3rd days of fermentation. The protease activity was not detected prior to 1st day, however the activity was started increase after 1st day of fermentation (0.126U/mg) and then increase with the fermentation time till it reached a maximum at 3rd days (2.62 U/mg), and then decrease again (Fig. 1). The similar pattern was also reported in pure culture induced *kinema* fermentation from indian and Canadian varieties of soybean [44] and also in *thua-nao* natural fermentation [45]. However, the fermentation time when proteolysis reach the highest level has shown to be different even in kinema prepared from Indian variety(22h) and Canadian variety (46h) [44]. Similarly, during *thua-nao* fermentation the optimum activity was reported to reach in 60h [45]. Generally, proteolysis in natural soybean fermentation reached to optimum level slightly delay as compared to pure culture fermentation. The proteolytic activity during the soybean fermentation depends on many factors associated with sources of microorganism such as ingredients, equipment, and wrapping materials [25]. The delay in appearing of the proteolytic band in natural fermentation compared to the pure culture fermentation of *thua-nao* was well reported [46]. In many alkaline soybean fermented products, *B. subtilis* has been shown to increase with fermentation [28,45]. *B. subtilis* grew on soybean surface release the proteases that break down soy proteins, increases the protein concentration and proteolytic activity during the *thua-nao* fermentation [46]. So, it could be expected that *B. subtilis* had an important role on secretion of exoprotease during *kinema* fermentation also, and was responsible for rising the activity maximum after 3rd day of fermentation.

### 3.3 Partial purification of crude enzyme

The extracted crude protease from *kinema* was precipitated with a gradual increase of 0–80% ammonium sulfate saturation level. The fractions obtained at 30–40%, 40–50 %, 50–60% and 60–70% retained comparatively higher protease activity and specific activity than other lower or higher fractions (Table 2). The activity of the protease increase or decrease due to variation in the amount of enzymatic or non-enzymatic protein that settles in each fractions and their solubility at the respective salt concentrations [47].

**Table 2:** Ammonium sulfate precipitation of protease from *kinema*.

Ammonium Sulfate Saturation (%)	Volume (ml)	Protease Activity (PA) (U/ml)	Protein (mg/ml)	Total PA (U)	Total Protein (mg)	Specific Activity) (U/mg)
Crude	10	0.532 ± 0.025	0.164 ± 0.006	5.318	1.640	3.243
0–20	2	0.038 ± 0.005	0.222 ± 0.000	0.076	0.443	0.171
20–30	2	0.100 ± 0.008	0.113 ± 0.032	0.201	0.225	0.892
30–40	2	0.512 ± 0.013	0.076 ± 0.001	1.025	0.151	6.765
40–50	2	0.624 ± 0.008	0.051 ± 0.004	1.248	0.102	12.269
50–60	2	0.614 ± 0.003	0.101 ± 0.002	1.227	0.202	6.066
60–70 %	2	0.619 ± 0.042	0.081 ± 0.001	1.237	0.162	7.650
70–80%	2	0.094 ± 0.005	0.039 ± 0.006	0.187	0.078	2.390
80% (supernatant)	14	0.022 ± 0.026	0.026 ± 0.001	0.315	0.364	0.865

Accordingly, the range 30–70% was selected for precipitation of enzyme for further purification by dialysis. After dialysis, specific activity was found increase to 7.90 U/mg with an increase in purification fold to 2.45 and recovery of 63.21 % of total activity (Table 3). The purification factor and % yield of the *kinema* protease obtained in this study is within the ranges of 1.67–4.89 and 9.25%–59.75% respectively as reported by several researchers [18,21,48,49].

**Table 3:** Partial purification of protease from *kinema*.

Purification Steps	Total Volume (ml)	Protease activity (U/ml)	Protein (mg/ml)	Total Activity (U)	Total Protein (mg)	Specific Activity (U/mg)	Purification fold	Yield %
Crude	100	0.508	0.158	50.791	15.770	3.22	1.00	100.00
30–70% Amm. sulf.	15	1.939	0.377	29.092	5.651	5.15	1.60	57.28
Dialysed	18.5	1.735	0.220	32.105	4.062	7.90	2.45	63.21

For the kinetic analysis and characterization of the protease, several researchers have previously used partially purified protease extract obtained by ammonium sulfate precipitation and dialysis [[50], [51], [52]]. Shaikh et al. [51]mentioned an increase of protease activity to 0.83U/ml from 0.3 U/ml with similar purification steps. Sahin et al. [50] reported 6.4 fold purification with 35% yield after dialysis of 40–80% ammonium sulfate fraction of crude supernatant of *Bacillus subtilis* protease. Thomas et al. [52] obtained 6 fold purification with yield of 34% after dialysis of 60–80% ammonium sulfate fractions of crude supernatant obtained from *Bacillus Sp.*, TSA5 strains. It has been mentioned that 40–80% ammonium sulfate saturation provide the best effect for concentration of the protease and for removal of the unwanted proteins presence in the crude extract [53].

### 3.4 Effect of pH on activity of the *kinema* protease

**Fig. 2:**
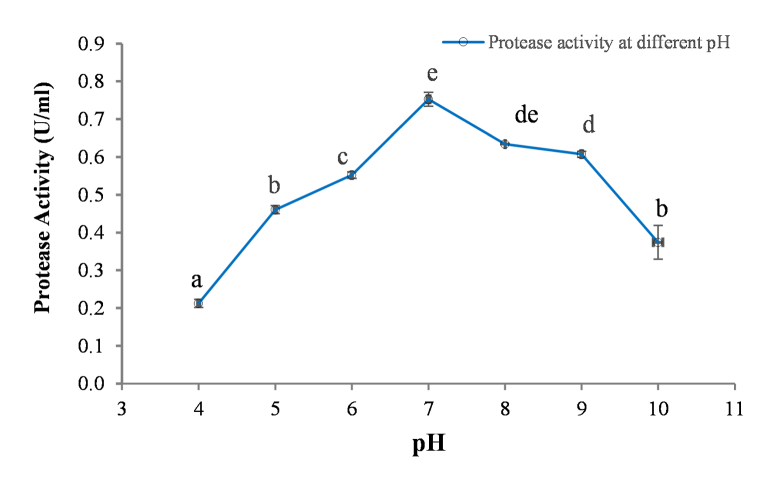
Effect of pH on activity of the *kinema* protease. Values are mean of triplicate ± SD, Mean value with different alphabet are significantly different at 5% level of significance (p < 0.05); protease activity was assayed on casein solution (5 mg/ml)) using the appropriate buffer system and adjusted to pH 4–10.

The protease activity was gradually increased up to pH 7.0 and then start decreasing (Fig. 2). The activity observed at pH 7 was significantly different with the activity obtained at pH values of 4, 5, 6, 9 and 10 but not significantly different with pH 8 (Fig. 2). This shows that optimal pH range of *kinema* protease was 7–8, however it remains active in a broad alkaline range (pH 6 to pH 9). *B. subtilis* is the predominant microorganism in *kinema* [54]. Proteases from *B. subtilis* have been categorized into neutral and alkaline proteases, with the former having ideal pH of 7 and the latter having pH optimal range of 9–11 [13]. The amino acid composition play influencing role in determining optimal pH values of protease, and an earlier works on nattokinase demonstrated that and acidic situation caused the activity to decline more quickly than an alkaline condition [11]. Similarly, It has also been shown that various *Bacillus* spp. were able to grow within pH level of 7–12 with better protease production which also supports the findings of this study [55].

The optimum pH and stability to moderate alkaline condition (pH 6–9) showed the *kinema* protease most resembled with commercially available neutrase and alcalase from *Bacillus* spp [2,7,56]. Neutrase and alcalase from *Bacillus* spp were mentioned to have potential use in food processing and food protein hydrolysis [7,57], wort processing in brewery [58], detergent and leather processing because of dehairing and destaining capability [[59], [60], [61]], animal waste treatment and management due to their keratinolytic activities [[62], [63], [64]]. Similarly many fibrinolytic enzymes from the *Bacillus* species had been reported to have similar pH stability [[65], [66], [67]].

### 3.5 Effect of temperature and temperature stability of the *kinema* protease

**Fig. 3:**
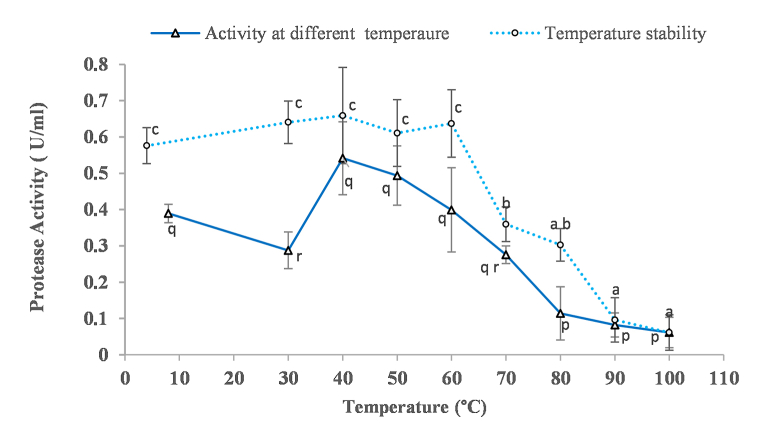
Effect of temperature on activity and stability of the *kinema* protease. *Value are mean of triplicate ± SD, Mean value with different alphabet are significantly different at 5% level of significance (p<0.05)*.

The protease activity of *kinema* protease increased from 30 °C to 40 °C and then started to decrease gradually (Fig. 3). The protease activity at 40 °C was not significantly different from the activity at temperatures 50 °C and 60 °C, but found significantly different with activity on other tested temperatures (Fig. 3). This reflect that the optimum range of *kinema* protease lies between 40 and 60 °C. Regarding temperature stability, the enzyme was found stable and retained the similar activity from 4 to 60 °C (Fig. 3). As the enzyme was treated above 60 °C, the protease activity rapidly decreased, although some activity was observed even after 80 °C treatment. These finding imply that protease obtained from *kinema* could be considered as a thermostable [68]. Gençkal and Tari [69] reported that the drop in proteolytic performance above 60 °C is because of heat-induced denaturation. Similar ideal temperature (40 °C) was also observed with *B. subtilis*-nattokinase from *natto*, a product similar to *kinema* [11]. Several researchers have also reported the optimal activity in between 45 °C and 50 °C for protease produced from other strains of *Bacillus* [12,21,55]. For use in leather processing and detergent, thermostability was mentioned to be crucial characteristics of the proteases [70].

### 3.6 Kinetic parameter of the *kinema* protease

**Fig. 4:**
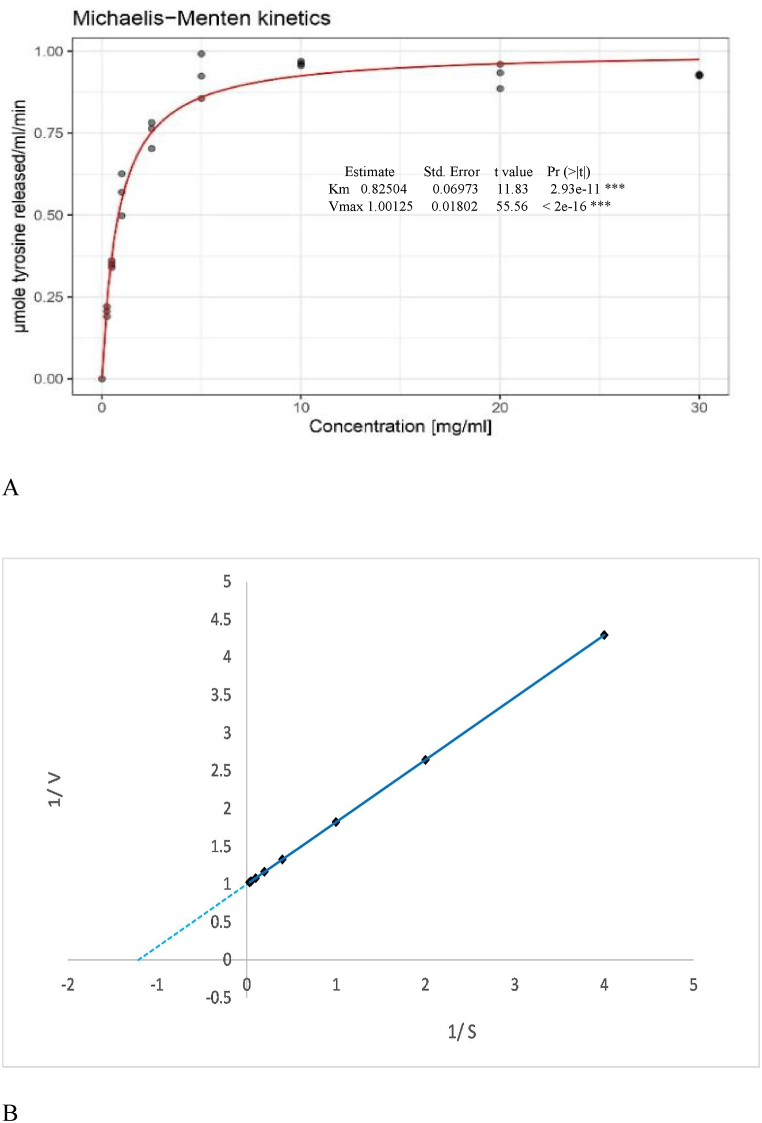
(a): Michaelis-Menten plot for the *kinema* protease activity of as a function of substrate concentration (b): Line-Weaver Burk plot for the *kinema* protease activity of as a function of substrate concentration.

The kinetic parameters (K_m_ and V_max_) were estimated by conducting a kinetic study of the *kinema* protease with increasing casein concentrations (0.25–30 mg/ml), keeping the temperature and pH constant (Fig. 4a &Fig. 4b). K_m_ implies the enzyme affinity towards its substrate; a low value indicates high substrate affinity, and V_max_ represents the higher reaction rate of an enzyme at its saturation level. After fitting data into the Michaelis-Menten curve and Lineweaver-Burk graph, K_m_ and V_max_ were found as 0.82 mg/ml and 1.001 μmole tyrosine released/ml/min (181.37 μg tyrosine/ml/min) respectively (Fig. 4a & b). The K_m_ and V_max_ values can be varied with environmental conditions, substrates and applied assay methods [71]. The K_m_ and V_max_ obtained in this study were within the ranges of K_m_ (0.25–2.3 mg/ml) and V_max_ (148–473 μg tyrosine/ml/min respectively) values reported previously for neutral and alkaline proteases from *Bacillus* [48,49,72]. For alkaline protease obtained from *B. cereus* isolate, K_m_ and V_max_ on casein were reported as 0.25 mg/ml and 310 U/ml respectively [49]. Similarly the K_m_ and V_max_ with substrate p-nitrophenyl acetate for *B. subtilis* protease were stated to be 0.43 mM and 12000U/mg [53]. Iqbal et al. [73] has shown the K_m_ and V_max_ values on casein as 0.03064 μM (0.75 μg/ml) and 69.76 U/ml) for UV mutant, and 0.02669 μM (0.65 μg/ml) and 56.73 U/ml for native protease obtained from *B. subtilis*.

Higher Km value obtained in the present study as compared to those researcher [48,49,73] is most probably due to the less purification fold, which could also be evidenced from SDS page analysis (Fig. 9a). The inhibitors or activator or mixture of enzymes if presents can interact with the enzymatic performance and influences kinetic parameters [74]. It has been shown that crude enzyme extract required more free activation energy and less deactivation energy to bind the substrate compared to pure enzymes indicating that purification is quite important to improve the enzyme affinity to the substrate [75,76]. It reflects that further purification and separation of the enzyme to the homogeneity level; applying chromatography and gel filtration techniques could be advantageous to improve the substrate affinity and getting the lower Km values.

### 3.7 Effect of protease inhibitors

**Fig. 5:**
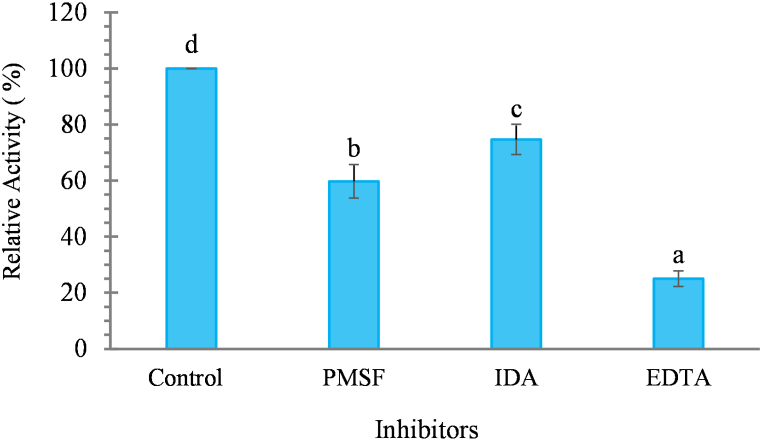
Effect of protease inhibitors (5 mM concentration) on activity of the *kinema* protease. *Value are mean of triplicate ± SD, Mean value with different alphabet are significantly different at 5% level of significance (p<0.05); enzyme extract was first treated with the inhibitors* 10 mM *concentration of the sodium phosphate buffer (1:1 v/v), incubated at 37°C for 1h and residual activity was determined by performing protease assay.*

The influence of inhibitors such as EDTA, IDA and PMSF on partially purified *kinema* protease activity is presented in Fig. 5. The protease activity with EDTA was found to be less which suggests that the highest inhibition effect has obtained by EDTA (74.99%) followed by PMSF (40.25%). These findings indicated that protease from *kinema* could be a metal-dependent serine protease or consist of a mixture of these types of proteases. Mamo and Assefa [77] also reported that most of the commercial neutral and alkaline serine proteases were obtained from bacteria of the genus *Bacillus. Bacillus* spp.; mainly *B. subtilis* predominated in the later phase of both natural and control fermentation of *kinema* also [28,29]. The finding about the inhibition of *kinema* protease by EDTA and PMSF in this study is in accordance with the study carried out with extracellular protease produced from *B. subtilis* and *B. licheniformis* isolated from crustacean wastes and polluted water [78,79].

### 3.8 Specificity toward natural substrates

**Fig. 6:**
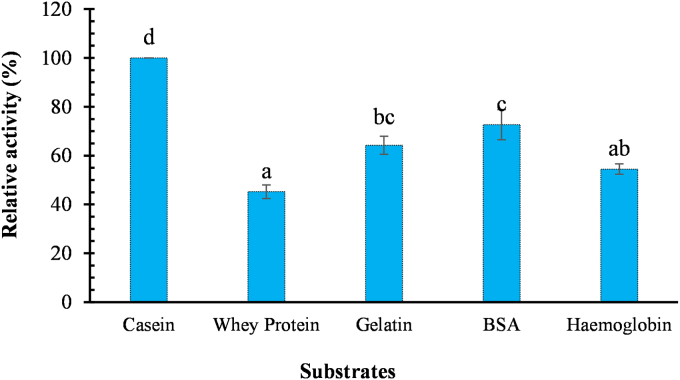
Activity of the *kinema* protease to different natural substrates. *Value are mean of triplicate ± SD, Mean value with different alphabet are significantly different at 5% level of significance (p<0.05); protease activity were performed with different natural substrate at the same concentration* 5/mg ml *and the result were expressed in term of relative activity considering the activity in casein as 100%.*

When assayed with different natural substrates, *kinema* protease showed the highest activity on casein followed by BSA, gelatin, hemoglobin and the whey protein (Fig. 6). It reflect that partially purified *kinema* protease had broad substrate specificity but had different catalyzing activity depending on the substrates. Microbial serine proteases have a wide range of natural and synthetic substrates that they can cleave, according to Gupta et al. [80]; in many cases, casein exhibits the significantly higher activity compared to azocasein, hemoglobin and BSA. Casein, ovalbumin and BSA have been shown to be specifically acted by serine protease isolated from *B. circulans* M − 34, although some activity was reported on gelatin [81]. In addition, chicken albumin was also reported as a good substrates for serine proteases obtained from *B. Subtilis* EAG-2 [82]. The capability of *kinema* protease to cleave varieties of proteins substrates; emphasized its applicability in enzymatic protein hydrolysate preparations from various food and animal proteins [57].

### 3.9 Effect of metal ions on *kinema* protease

**Fig. 7:**
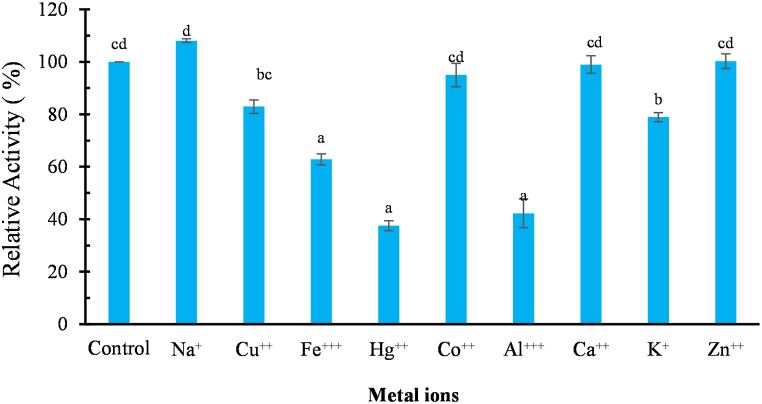
Effect of metal ions on activity of the *kinema* protease. *Value are mean of triplicate ± SD, Mean value with different alphabet are significantly different at 5% level of significance (p<0.05); metal ion were dissolved at the concentration of* 5 mM *in the reaction mixture, incubated for* 15 min *at 40°C and relative activity was measured compared to the control (reaction mixture with no added metal ions)*.

Sodium had enhancing effect whereas mercury, aluminum and iron displayed inhibition on activity of the protease (Fig. 7). Sodium enhanced the protease activity by 8% while calcium, cobalt and zinc have relatively similar activities as compared to control (Fig. 7). It has been reported that monovalent Na^+^ ions assist in refolding, and participated in activity regulation of serine protease isolated from *Bacillus* spp [83]. Additionally, it was noted that enzymatic activity was completely limited by Hg^++^, improved in the presence of Na^+^ and retained almost entirely in the presence of Zn^++^ [83]. Wang et al. [11] also found activation by Zn^++^ but inhibition by Al^+++^ and Fe^+++^ in purified nattokinase isolated from *natto*, a product similar to *kinema*. Metal ion has an essential role in enzymatic activity and enzyme production; *B. subtilis* when grown on NaCl and ZnCl_2_ has been shown to increase protease production but in contrast KCl reduced the production [84]. Similarly, divalent metal ions such as cobalt and zinc were stated to increase the activity in many commercial neutrase and alkaline protease from microbial sources too [3]. The results indicate that divalent metal ions such as Ca^++^, Na^+^, and Zn^++^ could play a potential application in enhancing the structural and thermal stability of the *kinema* protease. Metal ions can function as ion bridge to preserve the structure integrity or stabilize enzyme -substrate complex, prevent from autolysis and thermal unfolding [70].

### 3.10 Storage stability of *kinema* protease

**Fig. 8:**
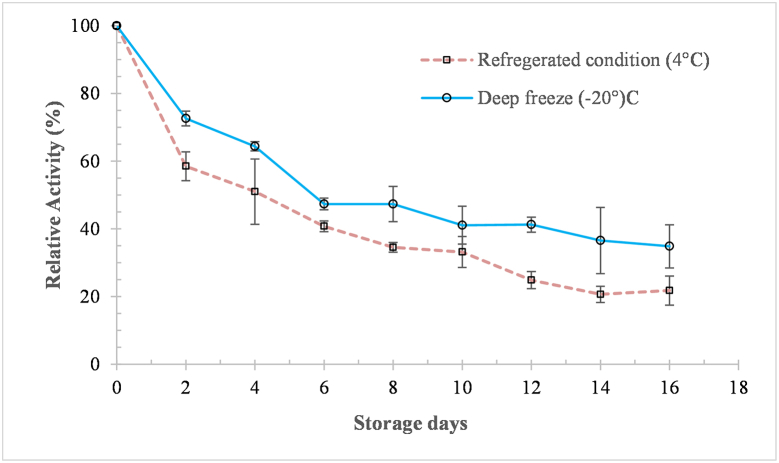
Storage stability of the *kinema* protease. *Value are mean of triplicate ± SD*.

Protease stability is a crucial factor that significantly determines its utility and commercial feasibility. Understanding the durability and longevity of the enzyme’s functionality is crucial for its industrial manufacturing. The stability of the *kinema* protease was determined by storing at two distinct temperatures (−20 °C and 4 °C) for 16 days. The findings are depicted in Fig. 8. The loss in activity was found comparatively less and slower in enzyme stored at −20 °C than at 4 °C during sixteen days of storage (Fig. 8). About 50% loss in activity was found on the 6th and 4th day of storage at −20 °C and 4 °C respectively. The activity retained at −20 °C and 4 °C storage in 16th days were 34.85% and 21.79% respectively (Fig. 8). A neutral protease from *B. subtilis* KIBGE-HAS has been shown to lose its activity within ten days at refrigerated conditions [85]. The loss in activity even in the commercial enzyme during storage conditions is a common process and mainly attributed to covalent (oxidation, auto-digestion and racemization), and non-covalent (precipitation, aggregation, denaturation) modifications [86].

**Fig. 9:**
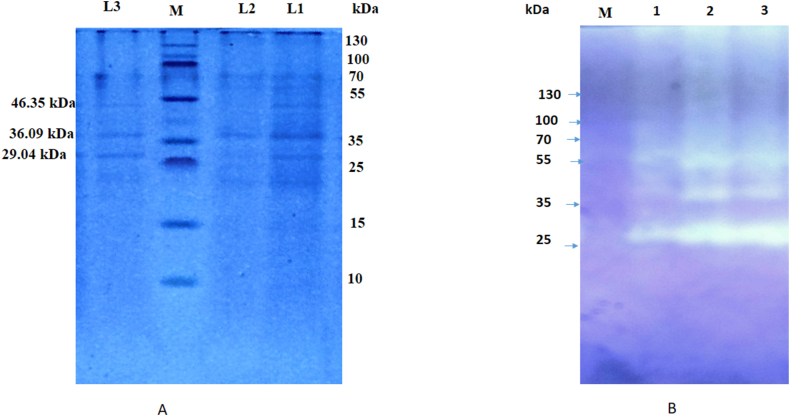
Electrophoretic analysis of *kinema* protease extract: (A) SDS-PAGE (M, protein marker; L_1_, crude kinema extract; L_2_, 30–70 % ammonium sulfate precipitate; L_3_, dialysed kinema protease at concentration of 10 μg) (B) Gelatin zymogram of dilalysed kinema protease extract (1 at 5 μg; 2 at 7 μg and 3 at 10 μg loaded concentrations) (see supplementary_Fig. 9 A&B).

### 3.11 SDS-PAGE and zymography analyses

Tricine SDS-PAGE analysis under reducing condition showed that dialyzed *kinema* protease consist of three bands reflecting apparent molecular weight (Mr) of 29.04 KDa, 36.09 kDa and 46.35 kDa respectively (Fig. 9a). The corresponding zymography showed all the three band degraded the gelatin, and the activity was increased with increased loaded concentration. Among them, the most intensive band was observed with 29.04 kDa suggesting the most active proteinase fractions presence in the existing protease extract (Fig. 9b). *Baciilus* protease has been reported to encode major eight extracellular protease (five serine proteases and three metalloproteases) generally at stationary phase of the fermentation. Among them protease aprE (Serine protease, Subtilisin family S_8_) and NprE (Metalloprotease; family M_4_) are the major protease representing in the fermentation broth [87]. These proteases can proceed to autolysis or induced lysis to different fragmented active forms having variants in the apparent molecular weights. Hence obtaining the multiple active band in the partially purified *kinema* protease extract could be possible. The multiple bands have also been observed previously with partially purified and crude protease extract obtained from *B. subtilis* G_8_ isolate from *natto* [88,89].

The molecular weight of the proteases obtained from different fermented soybean foods or their isolates (*Bacillus* sp. *and B. subtilisin*) were reported to be ranges from 21 kDa to 45 kDa and were mostly the subtilisin like serine proteases or metal dependent serine proteases or metalloproteases [9]. The active proteinase fraction (Mr 29.04 kDa) obtained in the present study was arguably close to molecular weight of serine proteases such as Nattokinase (Mr 28 kDa) [17] and nattoprotease (Mr 29 kDa) [18] from *natto*; *B. subtilis* Qk-2 (Mr 28 kDa) from fermented soybean [90]; *B. subtilisin* DJ4 (29 kDa) from *Doen-jang* [91]; *Bacillus* sp. CK (Mr 28.2 kDa) from *Chungkook-jang* [23], *B. amyloliquefaciens* DC-4 (Mr 28 kDa; subtilisinDFE) from *meju* [22], *B. subtilis* DC27 (DEF27; Mr 29 kDa) from *douchi* [21] and metalloproteases such as *B. subtilisin* B-12 nattokinase (Mr 29 kDa) [11]. The band (Mr 36.09 kDa) obtained in this study was more close to the fibrinolytic serine protease (36.2 kDa) obtained from TH-5 strain isolated from *black tauco,* an Indonesian soybean paste [92] and near to neutral metalloprotease NPR68 (Mr 32.7 kDa) from *meju* [24]. The other band (Mr 46.35 kDa) was slightly higher than ranges of earlier reported subtilisin and subtilisin like serine proteases or fibrinolytic metalloproteases obtained fermented soybean foods [9,10]. However, it was found near to the fibrinolytic protease (Mr; 43–46 kDa) obtained from the *Bacillus* sp. *nov* SK006 isolate from the indigenous Asian seasoning; shrimp paste [65]. This enzyme was mentioned to be quite difference in N-terminal sequence as compared to the most of subtilisin and subtilisin like proteases [65]. Based on these discussion, it can be postulated that *kinema* protease extract consist of both subtilisin and subtilisin like serine proteases and metalloprotease; that may also supported by previously explained inhibitors results, where both EDTA and PMSF inhibited the activity (Fig. 5).

Moreover, 30 kDa serine proteases from various *B. subtilis* strains have demonstrated to possess destaining and dehairing properties [53] and degradation ability of keratineous substrates [62]. Similarly metalloprotease of 28.24 kDa [61], 32 kDa [64], 39 kDa [63] obtained from *B. subtilis* mentioned to have promising ability of applying in detergent, laundry and feather degradations. Crustacean waste has also been reported to be deproteinized by 44 kDa metalloprotease obtained from *B. subtilis* [79]. As well, the metalloprotease of 38 kDa from *B. subtilis* has reported to be applicable in food processing and food protein hydrolysates [57]. The active three bands found in this study were also close with molecular weights of the proteases mentioned above by the researchers. It showed that *kinema protease* could have possibilities to apply in industrial application too.

Collectively, these findings support the existence of the many proteases in *kinema* extract and the possibility that most of them have fibrinolytic activity or could have other industrial applications. However, to confirm further research regarding isolation of each protease and their purification at the homogeneity level, characterization and assessing their applicability for utilization is more important.

## 4 Conclusion and recommendations

The study revealed that protease activity during natural *kinema* fermentation reach at highest after 3 days of fermentation. The partial purification following dialysis after precipitation with ammonium sulfate can improve the purification of *kinema* protease by 2.45 fold with 63.21% of recovery of total activity. *Kinema* protease can withstand and remains active in wider temperatures and pH ranges, with optimal activity at 40–60 °C and 7–8 pH, and probably are metal dependent serine protease or mixture of them. A further study is warranted to confirm the type of the protease.

Overall, *kinema* could be an important source of proteases, and can be used as a source of protease and proteolytic strains for further study and utilization for several industrial applications in the upcoming future.

## CRediT authorship contribution statement

**Dambar Bahadur Khadka:** Supervision, Investigation, Conceptualization. **Tikaram Pahadi:** Investigation, Formal analysis. **Sunil Aryal:** Formal analysis, Data curation. **Dhan Bahadur Karki:** Writing – review & editing, Supervision, Project administration, Investigation, Conceptualization.

## Declaration of competing interest

The authors declare that they have no known competing financial interests or personal relationships that could have appeared to influence the work reported in this paper.

## References

[1] Allied A.M.R. (2023). http://www.alliedmarketresearch.com/enzymes-market.

[2] Razzaq A., Shamsi S., Ali A. (2019). Microbial protease applications. Front. Bioeng. Biotechnol..

[3] Singh R., Mittal A., Kumar M. (2016). Microbial proteases in commercial applications. J. Pharm. Chem. Biol. Sci.

[4] Ben Amira A., Makhlouf I., Flaviu Petrut R. (2017). Effect of extraction pH on techno-functional properties of crude extracts from wild cardoon (Cynara cardunculus L.) Flowers. Food Chem..

[5] Jabalia N., Mishra P.C., Chaudhary N. (2014). Application, challenge and future propspectives of protease: an overview. J. Agroecol. Nat. Resour. Manag..

[6] Mazarro-Manzano M.A., Ramirez-Suarez J.C., Yada R.Y. (2018). Plant protease for bioactive peptide release: a review. Crit. Rev. Food Sci. Nutr..

[7] dos Santos Aguilar J.G., Sato H.H. (2018). Microbial proteases: production and application in obtaining protein hydrolysates. Food Res. Int..

[8] Contesini F.J., Melo R.R., Sato H.H. (2017). An overview of Bacillus proteases: from production to application. Crit. Rev. Biotechnol..

[9] Peng Y., Yang X., Zhang Y. (2005). Microbial fibrinolytic enzymes: an overview of source, production, properties, and thrombolytic activity in vivo. Appl. Microbiol. Biotechnol..

[10] Wang P., Peng C., Xie X. (2023). Research progress on the fibrinolytic enzymes produced from traditional fermented foods. Food Sci. Nutr..

[11] Wang C., Du M., Zheng D. (2009). Purification and characterization of nattokinase from Bacillus subtilis natto B-12. J. Agric. Food Chem..

[12] Zhang X., Tong Y., Wang J. (2021). Screening of a Bacillus subtilis strain producing both nattokinase and milk-clotting enzymes and its application in fermented milk with thrombolytic activity. J. Dairy Sci..

[13] Chantawannakul P., Oncharoen A., Klanbut K. (2002). Characterization of proteases of Bacillus subtilis strain 38 isolated from traditionally fermented soybean in Northern Thailand. Sci. Asia.

[14] Afifah D.N., Sulchan M., Syah D. (2014). Purification and characterization of a fibrinolytic enzyme from Bacillus pumilus 2.g isolated from Gembus, an Indonesian fermented food. Preventive Nutr. Food Sci..

[15] Yao Z., Liu X., Shim J.M. (2017). Properties of a fibrinolytic enzyme secreted by Bacillus amyloliquefaciens RSB34, isolated from Doenjang. J. Microbiol. Biotechnol..

[16] Jeong S.J., Heo K., Park J.Y. (2015). Characterization of AprE176, a fibrinolytic enzyme from Bacillus subtilis HK176. J. Microbiol. Biotechnol..

[17] Fujita M., Nomura K., Hong K. (1993). Purification and characterization of a strong fibrinolytic enzyme (nattokinase) in the vegetable cheese natto, a popular soybean fermented food in Japan. Biochem. Biophys. Res. Commun..

[18] Kashihara K., Nio N., Kubota K. (2001). Purification and characterization of a protease in comercial Natto. Nippon Shokuhin Kagaku Kaishi.

[19] Dabbagh F., Negahdaripour M., Berenjian A. (2014). Nattokinase: production and application. Appl. Microbiol. Biotechnol..

[20] Sharma D., Shekhar S.K., Kumar A. (2020). Isolation, characterization, production and purification of fibrinolytic enzyme nattokinase from Bacillus subtilis. Int. J. Pharm. Sci. Res..

[21] Hu Y., Yu D., Wang Z. (2019). Purifcation and characterization of a novel, highly potent fbrinolytic enzyme from Bacillus subtilis DC27 screened from Douchi, a traditional Chinese fermented soybean food. Sci. Rep..

[22] Peng Y., Huang Q., Zhang R.-h. (2003). Purification and characterization of a fibrinolytic enzyme produced by Bacillus amyloliquefaciens DC-4 screened from douchi, a traditional Chinese soybean food. Comp. Biochem. Physiol. B Biochem. Mol. Biol..

[23] Kim W., Choi K., Kim Y. (1996). Purification and characterization of a fibrinolytic enzyme produced from Bacillus sp. strain CK 11-4 screened from Chungkook-Jang. Appl. Environ. Microbiol..

[24] Cho S.J., Oh S.H., Pridmore R.D. (2003). Purification and characterization of proteases from Bacillus amyloliquefaciens isolated from traditional soybean fermentation starter. J. Agric. Food Chem..

[25] Tamang J.P. (2015). Naturally fermented ethnic soybean foods of India. J. Ethn. Foods.

[26] Khadka D.B., Lama J.P., Prakash J., Waisundara V., Prakash V. (2020). Nutritional and Health Aspects of Food in South Asian Countries.

[27] Tamang J.P. (2003). Native microorganisms in the fermentation of kinema. Indian J. Microbiol..

[28] Sarkar P.K., Tamang J.P. (1995). Changes in the microbial profile and proximate composition during natural and controlled fermentations of soybeans to produce kinema. Food Microbiol..

[29] Sarkar P.K., Tamang J.P., Cook P.E. (1994). Kinema- a traditional soybean fermented food: proximate composition and microflora. Food Microbiol..

[30] Pandey G.R., Shrestha A., Karki T.B. (2022). Screening and identification of thermotolerant and osmotolerant Bacillus amyloliquefaciens BKHE isolated from kinema of eastern Nepal for alkaline protease production. Int. J. Microbiol..

[31] Bradford M.M. (1976). A rapid and sensitive method for the quantitation of microgram quantities of protein utilizing the principle of protein-dye binding. Anal. Chem..

[32] Cupp-Enyard C. (2008). Sigma's non-specific protease activity assay-casein as a substrate. J. Vis. Exp..

[33] AOAC (1990).

[34] Ranganna S. (1986).

[35] Nafi A., Foo H.L., Jamilah B. (2013). Properties of proteolytic enzyme from ginger (Zingiber officinale Roscoe). Int. Food Res. J..

[36] Abd-Eikhalek A.M., Seoudi D.M., Ibrahim O.A. (2020). Extraction, partial purification, characteristics, and antimicrobial activity of plant protease from Moringa Oleifera leaves. J. Appl. Biotechnol. Rep..

[37] Duong-Ly K.C., Gabelli S.B., Lorsch J. (2014). Laboratory Methods in Enzymology: Protein Part C.

[38] Sachin H.R., SharathKumar M.N., Devaraja S. (2021). Anticoagulant and antiplatelet activities of novel serine protease purified from seeds of Cucumis maderaspatensis. 3 Biotech.

[39] Adinarayana K., Ellaiah P., Prasad D. (2003). Purification and partial characterization of thermostable serine alkaline protease from a newly isolated Bacillus subtilis PE-11. AAPS PharmSciTech.

[40] Tomar R., Kumar R., Jagannadham M.V. (2008). A stable serine protease, wrightin, from the latex of the plant Wrightia tinctoria (Roxb.) R. Br.: purification and biochemical properties. J. Agric. Food Chem..

[41] Gagaoua M., Ziane F., Rabah S.N. (2017). Three phase partitioning, a scalable method for the purification and recovery of cucumisin, a milk-clotting enzyme, from the juice of Cucumis melo var. reticulatus. Int. J. Biol. Macromol..

[42] Laemmli U.K. (1970). Cleavage of structural proteins during the assembly of the head of bacteriophage T4. Nature.

[43] Bayero A.S., Datti Y., Adulhadi M. (2019). Proximate composition and the mineral contents of soya beans (Glycine max) available in Kano State, Nigeria. Chemsearch Journal.

[44] Sarkar P.K., Cook P.E., Owens J.D. (1993). Bacillus fermentation of soybeans. World J. Microbiol. Biotechnol..

[45] Chukeatirote E., Chainun C., Siengsubchart A. (2006). Microbiological and biochemical changes in thua-nao fermentation. Res. J. Microbiol..

[46] Visessanguan W., Benjakul S., Potachareon W. (2005). Accelerated proteolysis of soy proteins during fermentation of thua‐nao inoculated with Bacillus subtilis. J. Food Biochem..

[47] Scopes R.K. (1994). Protein Purification: Principles and Practice.

[48] Ahmed I., Zia M.A., Iqbal H.M.N. (2011). Purification and kinetic parameters characterization of an alakaline protease produced from Bacillus subtilis through submerged fermentation technique. World Appl. Sci. J..

[49] Lakshmi B.K.M., Kumar D.M., Hemalatha K.P.J. (2018). Purification and characterization of alkaline protease with novel properties from Bacillus cereus strain S8. J. Genet. Eng. Biotechnol..

[50] Sahin S., Demir Y., Ozmen I. (2020). Production of protease from Bacillus Subtilis under SSF and effect of organic solventson lyophilized protease preparations. Int. J. Chem. Res..

[51] Shaikh I.A., Turakani B., Malpani J. (2023). Extracellular protease production, optimization, and partial purification from Bacillus nakamurai PL4 and its applications. J. King Saud Univ. Sci..

[52] Thomas N.N., Archana V., Shibina S. (2020). Isolation and characterization of a protease from Bacillus sps. Mater. Today Proc..

[53] Uddin M.E., Ahmad T., Ajam M.M. (2017). Thermotolerant extracellular proteases produced by Bacillus subtilis isolated from local soil that representing industrial applications. J. Pure Appl. Microbiol..

[54] Sundus H., Mukhtar H., Nawaz A. (2016). Industrial applications and production sources of serine alkaline proteases: a review. J. Bacteriol. Mycol..

[55] Boominadhan U., Rajakumar R., Sivakumaar P.K. (2009). Optimization of protease enzyme production using Bacillus sp. isolated from different wastes. Bot. Res. Int..

[56] Matkawala F., Nighojkar S., Kumar A. (2021). Microbial alkaline serine proteases: production, properties and applications. World J. Microbiol. Biotechnol..

[57] Patel A.R., Mokashe N.U., Chaudhari D.S. (2019). Production optimisation and characterisation of extracellular protease secreted by newly isolated Bacillus subtilis AU-2 strain obtained from Tribolium castaneum gut. Biocatal. Agric. Biotechnol..

[58] Wang J., Xu A., Wan Y. (2013). Purification and characterization of a new metallo-neutral protease for beer brewing from Bacillus amyloliquefaciens SYB-001. Appl. Biochem. Biotechnol..

[59] Giri S.S., Sukumaran V., Sen S.S. (2011). Purification and partial characterization of a detergent and oxidizing agent stable alkaline protease from a newly isolated Bacillus subtilis VSG-4 of tropical soil. J. Microbiol..

[60] Rajkumar R., Jayappriyan K.R., Rengasamy R. (2011). Purification and characterization of a protease produced by Bacillus megaterium RRM2: application in detergent and dehairing industries. J. Basic Microbiol..

[61] Rehman R., Ahmed M., Siddique A. (2017). Catalytic role of thermostable metalloproteases from Bacillus subtilis KT004404 as dehairing and destaining agent. Appl. Biochem. Biotechnol..

[62] Ferrareze P.A.G., Correa A.P.F., Brandelli A. (2016). Purification and characterization of a keratinolytic protease produced by probiotic Bacillus subtilis. Biocatal. Agric. Biotechnol..

[63] Kokwe L., Nnolim N.E., Ezeogu L.I. (2023). Thermoactive metallo-keratinase from Bacillus sp. NFH5: characterization, structural elucidation, and potential application as detergent additive. Heliyon.

[64] Tork S.E., Shahein Y.E., El-Hakim A.E. (2013). Production and characterization of thermostable metallo-keratinase from newly isolated Bacillus subtilis NRC 3. Int. J. Biol. Macromol..

[65] Hua Y., Jiang B., Mine Y. (2008). Purification and characterization of a novel fibrinolytic enzyme from Bacillus sp. nov. SK006 isolated from an Asian traditional fermented shrimp paste. J. Agric. Food Chem..

[66] Kim H., Kim G., Kim D. (1997). Purification and characterization of a novel fibrinolytic enzyme from Bacillus sp. KA38 originated from fermented fish. J. Ferment. Bioeng..

[67] Wang S.L., Wu Y.Y., Liang T.W. (2011). Purification and biochemical characterization of a nattokinase by conversion of shrimp shell with Bacillus subtilis TKU007. N Biotechnol.

[68] Pant G., Prakash A., Pavani J.V.P. (2015). Production, optimization and partial purification of protease from Bacillus subtilis. J. Taibah Univ. Sci..

[69] Gençkal H., Tari C. (2006). Alkaline protease production from alkalophilic Bacillus sp. isolated from natural habitats. Enzym. Microb. Technol..

[70] Li Q., Yi L., Marek P. (2013). Commercial proteases: present and future. FEBS Lett..

[71] Bibi Z., Sahid F., Ul Qader S.A. (2015). Agar–agar entrapment increases the stability of endo-1,4-xylanase for repeated biodegradation of xylan. Int. J. Biol. Macromol..

[72] Kamran A., Bibi Z. (2015). Kinetic parameter analysis and pH stability of protease from thermophilic Bacillus species. Pakistan J. Biochem. Mol. Biol..

[73] Iqbal M., Asgher M., Bashir F. (2018). Purification and kinetic characterization of alkaline protease for UV-90 mutant of Bacillus Subtilis. J. Biochem. Analyt. Stud..

[74] Rogers A., Gibon Y., Schwender J. (2009). Plant Metabolic Networks.

[75] Braga A.R.C., Manera A.P., Ores J.C. (2013). Kinetics and thermal properties of crude and purified β-Galactosidase with potential for the production of galactooligosaccharides. Food Technol. Biotechnol..

[76] Soares da Silva O., Lira de Oliveira R., de Carvalho Silva J. (2017). Thermodynamic investigation of an alkaline protease from Aspergillus tamarii URM4634: a comparative approach between crude extract and purified enzyme. Int. J. Biol. Macromol..

[77] Mamo J., Assefa F. (2018). The role of microbial aspartic protease enzyme in food and beverage industries. J. Food Qual..

[78] Sellami-Kamoun A., Haddar A., Ali N.E.H. (2008). Stability of thermostable alkaline protease from Bacillus licheniformis RP1 in commercial solid laundry detergent formulations. Microbiol. Res..

[79] Yang J.K., Shih L., Tzeng Y.M. (2000). Production and purification of protease from a Bacillus subtilis that can deproteinize crustacean wastes. Enzym. Microb. Technol..

[80] Gupta R., Beg Q., Khan S. (2002). An overview on fermentation, downstream processing and properties of microbial alkaline proteases. Appl. Microbiol. Biotechnol..

[81] Sari E., Loğoğlu E., Öktemer A. (2015). Purification and characterization of organic solvent stable serine alkaline protease from newly isolated Bacillus circulans M34. Biomed. Chromatogr..

[82] Ghafoor A., Hasnain S. (2010). Purification and characterization of an extracellular protease from Bacillus subtilis EAG-2 strain isolated from ornamental plant nursery. Pol. J. Microbiol..

[83] Sinha R., Khare S.K. (2013). Characterization of detergent compatible protease of a halophilic Bacillus sp. EMB9: differential role of metal ions in stability and activity. Bioresour. Technol..

[84] Qureshi A.S., Bhutto M.A., Khushk I. (2011). Optimization of cultural conditions for protease production by Bacillus subtilis EFRL 01. Afr. J. Biotechnol..

[85] Anwar A., Qader S.A., Raiz A. (2009). Calcium alginate: a support material for immobilization of proteases from newly isolated strain of Bacillus subtilis KIBGE-HAS. World Appl. Sci. J..

[86] Misset O., van den Tweel A.H.W.J.J., Buitelaar R.M. (1993). Study in Organic Chemistry.

[87] Harwood C.R., Kikuchi Y. (2022). The ins and outs of Bacillus proteases: activities, functions and commercial significance. FEMS Microbiol. Rev..

[88] Lucy J., Raharjo P.F., Elvina E. (2019). Clot lysis activity of Bacillus subtilis G8 isolated from Japanese fermented natto soybeans. Appl. Food Biotechnol..

[89] Pinontoan R., Elvina A. Sanjaya (2021). Fibrinolytic characteristics of Bacillus subtilis G8 isolated from natto. Biosci. Microbiota. Food Health.

[90] Ko J.H., Yan J.P., Zhu L. (2004). Identification of two novel fibrinolytic enzymes from Bacillus subtilis QK02. Comp. Biochem. Physiol. C Toxicol. Pharmacol..

[91] Kim S.H., Choi N.S. (2000). Purification and characterization of subtilisin DJ-4 secreted by Bacillus sp. strain DJ-4 screened from Doen-Jang. Biosci. Biotechnol. Biochem..

[92] Yanti Screening (2018). purification, and characterization of fibrinolytic enzyme producing bacteria from Indonesian fermented foods. Scholars Acad. J. Biosci..

