# Supplementary material for: Partial purification and characterization of protease extracted from kinema
Source: Heliyon. 2024 Feb 27;10(5):e27173. doi: 10.1016/j.heliyon.2024.e27173 (PMC10923713; doi:10.1016/j.heliyon.2024.e27173)
Supplement: Multimedia component 1 [file mmc1.docx]

**Supplementary Informations**

1. Tyrosine Standard curve for determination of protease activity
2. BSA standard curve for Bradford assay for determination of protein
3. **Fermentation time and protease activity**
4. **Values of protein content, protease activity and specific activity at different fermentation time of *kinema***

| Fermentation time  (Day) | Protein (µg/m) | PA1 (U/ml) | PA2 (U/ml) | PA3 (U/ml) | SPA1 (U/mg) | SPA2  (U/mg) | SPA3  (U/ml) | Mean  SPA | SD |
| --- | --- | --- | --- | --- | --- | --- | --- | --- | --- |
| Day 0 | 55.93±7.90 | -0.026 | -0.032 | -0.020 | -0.469 | -0.567 | -0.366 | -0.467 | 0.101 |
| day1 | 69.12±13.73 | 0.005 | 0.012 | 0.021 | 0.074 | 0.173 | 0.311 | 0.186 | 0.119 |
| day 2 | 68.33±7.21 | 0.101 | 0.106 | 0.114 | 1.482 | 1.556 | 1.669 | 1.569 | 0.094 |
| Day 3 | 79.17±1.73 | 0.213 | 0.201 | 0.207 | 2.687 | 2.545 | 2.616 | 2.616 | 0.071 |
| Day4 | 87.99±14.06 | 0.211 | 0.193 | 0.220 | 2.394 | 2.193 | 2.505 | 2.364 | 0.158 |
| Day5 | 88.97±10.71 | 0.163 | 0.161 | 0.160 | 1.828 | 1.805 | 1.793 | 1.808 | 0.018 |
| Day6 | 83.38±8.67 | 0.082 | 0.079 | 0.100 | 0.981 | 0.944 | 1.198 | 1.041 | 0.137 |

| 1. **Tests of Normality** | | | | | | |
| --- | --- | --- | --- | --- | --- | --- |
|  | Kolmogorov-Smirnov^a^ | | | Shapiro-Wilk | | |
|  | Statistic | df | Sig. | Statistic | df | Sig. |
| SPActivity | .138 | 21 | .200^*^ | .919 | 21 | .083 |
| *. This is a lower bound of the true significance. | | | | | | |
| a. Lilliefors Significance Correction | | | | | | |


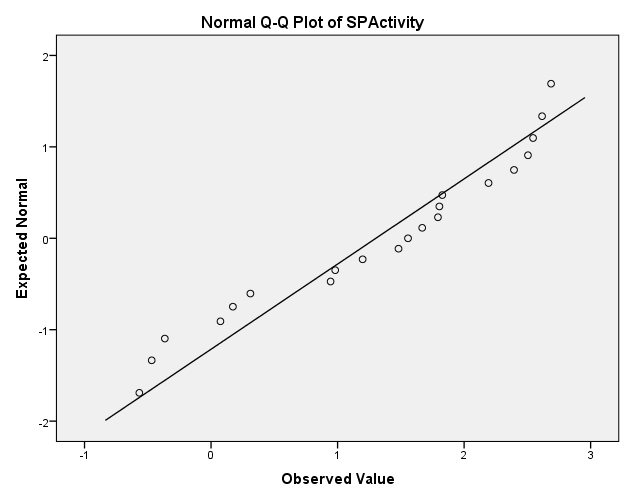

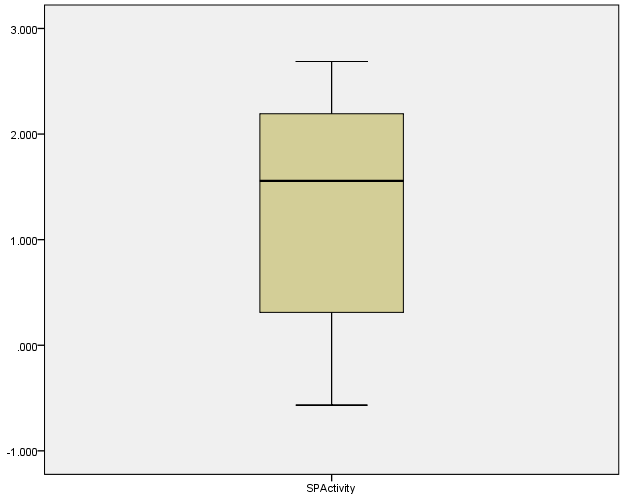


| 1. **Test of Homogeneity of Variances** | | | |
| --- | --- | --- | --- |
| SPActivity | | | |
| Levene Statistic | df1 | df2 | Sig. |
| 1.322 | 6 | 14 | .311 |

| 1. **ANOVA** | | | | | |
| --- | --- | --- | --- | --- | --- |
| SPActivity | | | | | |
|  | Sum of Squares | df | Mean Square | F | Sig. |
| Between Groups | 22.879 | 6 | 3.813 | 324.531 | .000 |
| Within Groups | .164 | 14 | .012 |  |  |
| Total | 23.043 | 20 |  |  |  |

1. **Homogeneous Subsets obtained from Post hoc test (TuKey HSD)**

| **SP Activity** | | | | | | |
| --- | --- | --- | --- | --- | --- | --- |
| Tukey HSD | | | | | | |
| FermTime | N | Subset for alpha = 0.05 | | | | |
|  |  | 1 | 2 | 3 | 4 | 5 |
| Day0 | 3 | -.46728 |  |  |  |  |
| Day1 | 3 |  | .18578 |  |  |  |
| Day6 | 3 |  |  | 1.04116 |  |  |
| Day2 | 3 |  |  |  | 1.56895 |  |
| Day5 | 3 |  |  |  | 1.80849 |  |
| Day4 | 3 |  |  |  |  | 2.36394 |
| Day3 | 3 |  |  |  |  | 2.61593 |
| Sig. |  | 1.000 | 1.000 | 1.000 | .167 | .133 |
| Means for groups in homogeneous subsets are displayed. | | | | | | |
| a. Uses Harmonic Mean Sample Size = 3.000. | | | | | | |

1. **Effect of pH on *kinema* protease activity**
2. **Values of protease activity at different pH**

| **pH** | **PA1  (U/ml)** | **PA2  (U/ml)** | **PA3  (U/ml)** | **Mean  PA (U/ml)** | **SD** |
| --- | --- | --- | --- | --- | --- |
| 4 | 0.201 | 0.223 | 0.213 | 0.212 | 0.016 |
| 5 | 0.450 | 0.471 | 0.461 | 0.461 | 0.015 |
| 6 | 0.543 | 0.560 | 0.553 | 0.551 | 0.008 |
| 7 | 0.733 | 0.770 | 0.755 | 0.751 | 0.019 |
| 8 | 0.633 | 0.636 | 0.634 | 0.634 | 0.002 |
| 9 | 0.599 | 0.614 | 0.610 | 0.606 | 0.011 |
| 10 | 0.420 | 0.331 | 0.372 | 0.375 | 0.063 |

| 1. **Tests of Normality** | | | | | | |
| --- | --- | --- | --- | --- | --- | --- |
|  | Kolmogorov-Smirnov^a^ | | | Shapiro-Wilk | | |
|  | Statistic | df | Sig. | Statistic | df | Sig. |
| PA | .140 | 21 | .200^*^ | .940 | 21 | .216 |
| *. This is a lower bound of the true significance. | | | | | | |
| 1. Lilliefors Significance Correction | | | | | | |


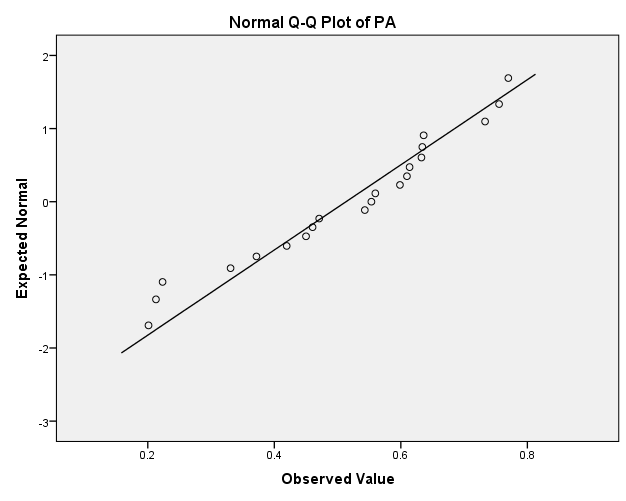

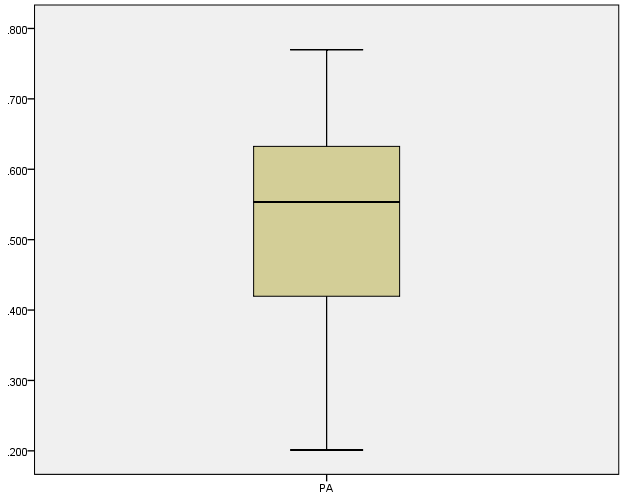


| 1. **Test of Homogeneity of Variances** | | | |
| --- | --- | --- | --- |
| PA | | | |
| Levene Statistic | df1 | df2 | Sig. |
| 2.470 | 6 | 14 | .077 |

| 1. **ANOVA** | | | | | |
| --- | --- | --- | --- | --- | --- |
| PA | | | | | |
|  | Sum of Squares | df | Mean Square | F | Sig. |
| Between Groups | .585 | 6 | .097 | 254.189 | .000 |
| Within Groups | .005 | 14 | .000 |  |  |
| Total | .590 | 20 |  |  |  |

1. **Homogeneous Subsets obtained from Post hoc test (TuKey HSD)**

| **PA** | | | | | | | |
| --- | --- | --- | --- | --- | --- | --- | --- |
| Tukey HSD | | | | | | | |
| pH | N | Subset for alpha = 0.05 | | | | | |
|  |  | 1 | 2 | 3 | 4 | 5 | 6 |
| 4 | 3 | .21246 |  |  |  |  |  |
| 10 | 3 |  | .37405 |  |  |  |  |
| 5 | 3 |  |  | .46051 |  |  |  |
| 6 | 3 |  |  |  | .55206 |  |  |
| 9 | 3 |  |  |  |  | .60732 |  |
| 8 | 3 |  |  |  |  | .63413 |  |
| 7 | 3 |  |  |  |  |  | .75273 |
| Sig. |  | 1.000 | 1.000 | 1.000 | 1.000 | .640 | 1.000 |
| Means for groups in homogeneous subsets are displayed. | | | | | | | |
| a. Uses Harmonic Mean Sample Size = 3.000. | | | | | | | |

**5. Effect of Temperature on *Kinema* protease activity and temperature stability**

**5.1 Effect of temperature**

1. Values of Protease activity at different temperature (Reaction temperature)

| Reaction  Temperature  (°c) | PA1  (U/ml) | PA2  (U/ml) | PA3  (U/ml) | Mean  PA(U/ml) | STD |
| --- | --- | --- | --- | --- | --- |
| 9 | 0.366 | 0.416 | 0.386 | 0.389 | 0.025 |
| 30 | 0.230 | 0.325 | 0.308 | 0.288 | 0.051 |
| 40 | 0.531 | 0.447 | 0.647 | 0.542 | 0.101 |
| 50 | 0.582 | 0.421 | 0.479 | 0.494 | 0.082 |
| 60 | 0.470 | 0.265 | 0.463 | 0.399 | 0.116 |
| 70 | 0.302 | 0.253 | 0.273 | 0.276 | 0.024 |
| 80 | 0.198 | 0.071 | 0.072 | 0.114 | 0.073 |
| 90 | 0.053 | 0.111 | 0.053 | 0.082 | 0.033 |
| 100 | 0.058 | 0.064 | 0.134 | 0.061 | 0.042 |

| 1. **Tests of Normality** | | | | | | |
| --- | --- | --- | --- | --- | --- | --- |
|  | Kolmogorov-Smirnov^a^ | | | Shapiro-Wilk | | |
|  | Statistic | df | Sig. | Statistic | df | Sig. |
| PA | .117 | 27 | .200^*^ | .943 | 27 | .142 |
| *. This is a lower bound of the true significance. | | | | | | |
| a. Lilliefors Significance Correction | | | | | | |


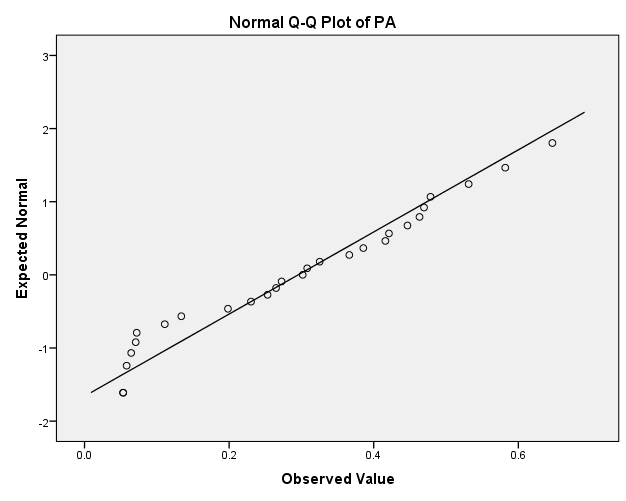

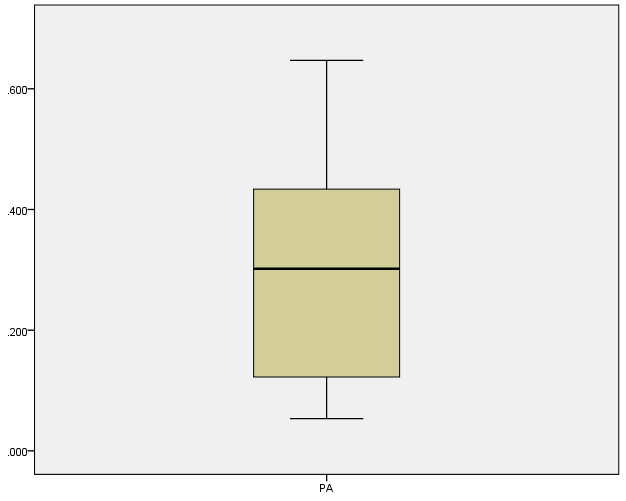


| 1. **Test of Homogeneity of Variances** | | | |
| --- | --- | --- | --- |
| PA | | | |
| Levene Statistic | df1 | df2 | Sig. |
| 2.331 | 8 | 18 | .065 |

| 1. **ANOVA** | | | | | |
| --- | --- | --- | --- | --- | --- |
| PA | | | | | |
|  | Sum of Squares | df | Mean Square | F | Sig. |
| Between Groups | .740 | 8 | .093 | 19.669 | .000 |
| Within Groups | .085 | 18 | .005 |  |  |
| Total | .825 | 26 |  |  |  |

1. **Homogeneous Subsets obtained from Post hoc test (TuKey HSD)**

| **PA** | | | | | | |
| --- | --- | --- | --- | --- | --- | --- |
| Tukey HSD | | | | | | |
| Temperature (ºC) | N | Subset for alpha = 0.05 | | | | |
|  |  | 1 | 2 | 3 | 4 | 5 |
| 90 | 3 | .07259 |  |  |  |  |
| 100 | 3 | .08554 | .08554 |  |  |  |
| 80 | 3 | .11374 | .11374 | .11374 |  |  |
| 70 | 3 |  | .27580 | .27580 | .27580 |  |
| 30 | 3 |  |  | .28782 | .28782 |  |
| 4 | 3 |  |  |  | .38931 | .38931 |
| 60 | 3 |  |  |  | .39925 | .39925 |
| 50 | 3 |  |  |  |  | .49381 |
| 40 | 3 |  |  |  |  | .54166 |
| Sig. |  | .997 | .061 | .106 | .442 | .208 |
| Means for groups in homogeneous subsets are displayed. | | | | | | |
| a. Uses Harmonic Mean Sample Size = 3.000. | | | | | | |

5**.2 Temperature Stability**

**i. Values of Protease activity of kinema protease treated with different temperatures**

| Temperature (°C) | Treatment  time | PA1  (U/ml) | PA2  (U/ml) | PA3  (U/ml) | Mean  PA (U/ml) | SD |
| --- | --- | --- | --- | --- | --- | --- |
| 4 | 30 min | 0.617 | 0.521 | 0.591 | 0.576 | 0.050 |
| 30 | 30 min | 0.701 | 0.584 | 0.637 | 0.641 | 0.059 |
| 40 | 30 min | 0.805 | 0.625 | 0.547 | 0.659 | 0.133 |
| 50 | 30 min | 0.508 | 0.639 | 0.686 | 0.611 | 0.092 |
| 60 | 30 min | 0.708 | 0.671 | 0.532 | 0.637 | 0.093 |
| 70 | 30 min | 0.385 | 0.304 | 0.390 | 0.360 | 0.048 |
| 80 | 30 min | 0.269 | 0.286 | 0.354 | 0.303 | 0.045 |
| 90 | 30 min | 0.081 | 0.111 | 0.198 | 0.096 | 0.061 |
| 100 | 30 min | 0.058 | 0.064 | 0.146 | 0.061 | 0.049 |

| 1. **Tests of Normality** | | | | | | |
| --- | --- | --- | --- | --- | --- | --- |
|  | Kolmogorov-Smirnov^a^ | | | Shapiro-Wilk | | |
|  | Statistic | df | Sig. | Statistic | df | Sig. |
| PA | .146 | 28 | .129 | .931 | 28 | .065 |
| a. Lilliefors Significance Correction | | | | | | |


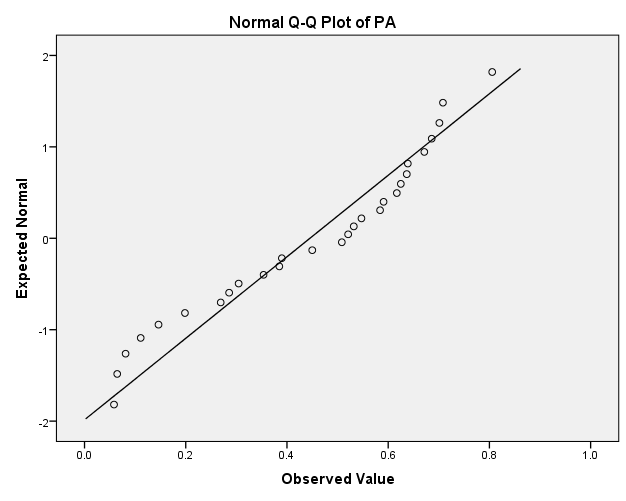

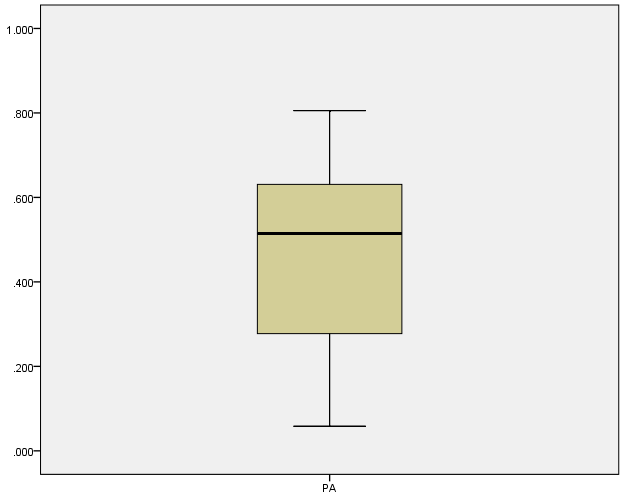


| 1. **Test of Homogeneity of Variances** | | | |
| --- | --- | --- | --- |
| PA | | | |
| Levene Statistic | df1 | df2 | Sig. |
| 1.465 | 8 | 18 | .237 |

| 1. **ANOVA** | | | | | |
| --- | --- | --- | --- | --- | --- |
| PA | | | | | |
|  | Sum of Squares | df | Mean Square | F | Sig. |
| Between Groups | 1.256 | 8 | .157 | 27.716 | .000 |
| Within Groups | .102 | 18 | .006 |  |  |
| Total | 1.358 | 26 |  |  |  |

1. **Homogeneous Subsets obtained from Post hoc test (TuKey HSD)**

| **PA** | | | | |
| --- | --- | --- | --- | --- |
| Tukey HSD | | | | |
| Temerature | N | Subset for alpha = 0.05 | | |
|  |  | 1 | 2 | 3 |
| 100 | 3 | .08963 |  |  |
| 90 | 3 | .13016 |  |  |
| 80 | 3 | .30285 | .30285 |  |
| 70 | 3 |  | .35972 |  |
| 4 | 3 |  |  | .57624 |
| 50 | 3 |  |  | .61101 |
| 60 | 3 |  |  | .63714 |
| 30 | 3 |  |  | .64061 |
| 40 | 3 |  |  | .65919 |
| Sig. |  | .053 | .988 | .902 |
| Means for groups in homogeneous subsets are displayed. | | | | |
| a. Uses Harmonic Mean Sample Size = 3.000. | | | | |

1. **Effect of Protease inhibitors**
2. **Values of protease activity and residual relative activity of protease in presence of protease inhibitors**

| Inhibitors | Concentration (mM) | PA1  (U/ml) | PA2  (U/ml) | PA3  (U/ml) | RRA1  (%) | RRA2  (%) | RRA 3  (%) | Mean  RRA (%) | SD |
| --- | --- | --- | --- | --- | --- | --- | --- | --- | --- |
| Control | 5 | 0.592 | 0.485 | 0.544 | 100.000 | 100.000 | 100.000 | 100.000 | 0.000 |
| PMSF | 5 | 0.342 | 0.267 | 0.362 | 57.768 | 54.993 | 66.497 | 59.752 | 6.003 |
| IDA | 5 | 0.408 | 0.367 | 0.433 | 68.815 | 75.668 | 79.490 | 74.658 | 5.409 |
| EDTA | 5 | 0.131 | 0.123 | 0.150 | 22.094 | 25.316 | 27.643 | 25.018 | 2.786 |

| 1. **Tests of Normality** | | | | | | |
| --- | --- | --- | --- | --- | --- | --- |
|  | Kolmogorov-Smirnov^a^ | | | Shapiro-Wilk | | |
|  | Statistic | df | Sig. | Statistic | df | Sig. |
| PA | .154 | 12 | .200^*^ | .904 | 12 | .176 |
| *. This is a lower bound of the true significance. | | | | | | |
| a. Lilliefors Significance Correction | | | | | | |


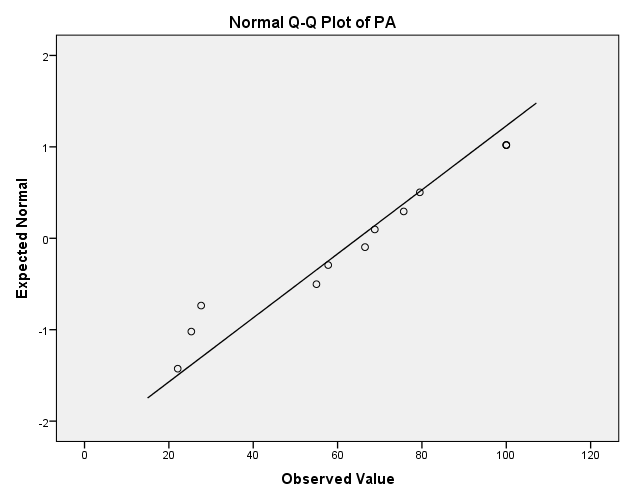

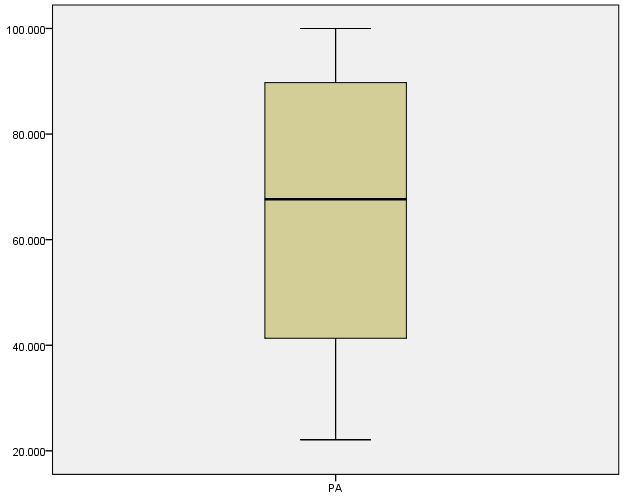


| 1. **Test of Homogeneity of Variances** | | | |
| --- | --- | --- | --- |
| PA | | | |
| Levene Statistic | df1 | df2 | Sig. |
| 3.490 | 3 | 8 | .070 |

| 1. **ANOVA** | | | | | |
| --- | --- | --- | --- | --- | --- |
| PA | | | | | |
|  | Sum of Squares | df | Mean Square | F | Sig. |
| Between Groups | 8832.852 | 3 | 2944.284 | 161.193 | .000 |
| Within Groups | 146.125 | 8 | 18.266 |  |  |
| Total | 8978.977 | 11 |  |  |  |

1. **Homogeneous subsets obtained from Post hoc test (TuKey HSD)**

| **PA** | | | | | |
| --- | --- | --- | --- | --- | --- |
| Tukey HSD | | | | | |
| inhibitor | N | Subset for alpha = 0.05 | | | |
|  |  | 1 | 2 | 3 | 4 |
| EDTA | 3 | 25.01804 |  |  |  |
| PMSF | 3 |  | 59.75244 |  |  |
| IDA | 3 |  |  | 74.65775 |  |
| Control | 3 |  |  |  | 100.00000 |
| Sig. |  | 1.000 | 1.000 | 1.000 | 1.000 |
| Means for groups in homogeneous subsets are displayed. | | | | | |
| a. Uses Harmonic Mean Sample Size = 3.000. | | | | | |

1. **Specificity toward natural substrates**
2. **Values of protease activity of kinema protease with different natural substrates**

| **Substrates** | **PA1  (U/ml)** | **PA2  (U/ml)** | **PA3  (U/ml)** | **RA1  (%)** | **RA2  (%)** | **RA3  (%)** | **Mean  RA (%)** | **SD** |
| --- | --- | --- | --- | --- | --- | --- | --- | --- |
| Casein (0.5%) | 0.592 | 0.585 | 0.588 | 100.000 | 100.000 | 100.000 | 100.000 | 0.000 |
| Whey protein (0.5%) | 0.254 | 0.283 | 0.261 | 42.907 | 48.341 | 44.458 | 45.235 | 2.799 |
| Gelatin (0.5%) | 0.361 | 0.399 | 0.373 | 60.961 | 68.246 | 63.467 | 64.225 | 3.701 |
| BSA (0.5%) | 0.394 | 0.462 | 0.426 | 66.589 | 78.910 | 72.406 | 72.635 | 6.164 |
| Haemoglobin (0.5%) | 0.311 | 0.332 | 0.319 | 52.521 | 56.754 | 54.198 | 54.491 | 2.132 |

| 1. **Tests of Normality** | | | | | | |
| --- | --- | --- | --- | --- | --- | --- |
|  | Kolmogorov-Smirnov^a^ | | | Shapiro-Wilk | | |
|  | Statistic | df | Sig. | Statistic | df | Sig. |
| PA | .152 | 15 | .200^*^ | .890 | 15 | .066 |
| *. This is a lower bound of the true significance. | | | | | | |
| a. Lilliefors Significance Correction | | | | | | |


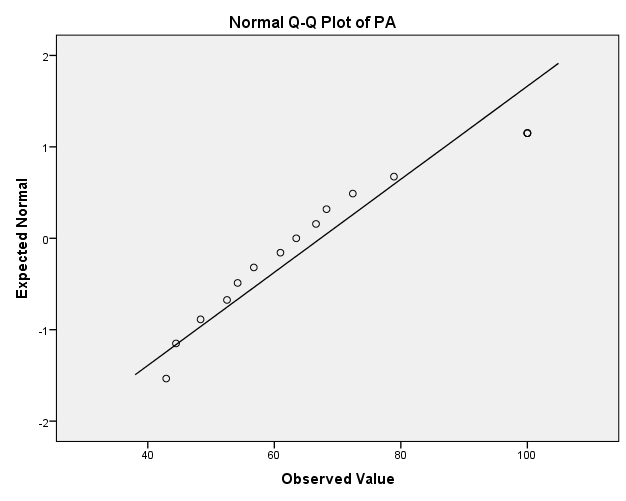

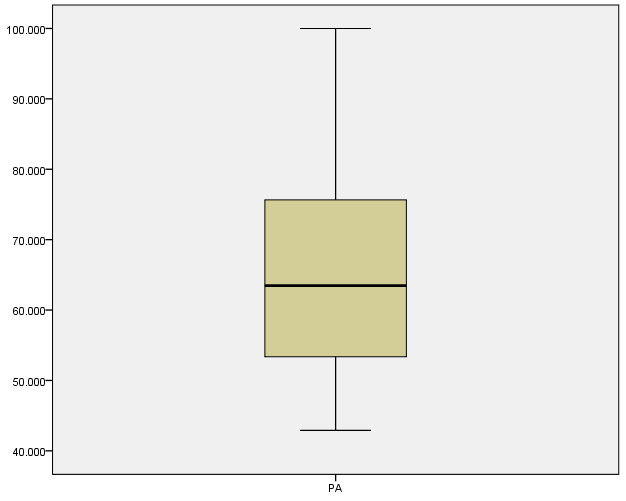


| 1. **Test of Homogeneity of Variances** | | | |
| --- | --- | --- | --- |
| PA | | | |
| Levene Statistic | df1 | df2 | Sig. |
| 2.058 | 4 | 10 | .162 |

| 1. **ANOVA** | | | | | |
| --- | --- | --- | --- | --- | --- |
| PA | | | | | |
|  | Sum of Squares | df | Mean Square | F | Sig. |
| Between Groups | 5274.374 | 4 | 1318.593 | 102.901 | .000 |
| Within Groups | 128.141 | 10 | 12.814 |  |  |
| Total | 5402.515 | 14 |  |  |  |

**v. Homogeneous subsets obtained from Post hoc test (TuKey HSD)**

| **PA** | | | | |
| --- | --- | --- | --- | --- |
| Tukey HSD | | | | |
| Ssubstrate | N | Subset for alpha = 0.05 | | |
|  |  | 1 | 2 | 3 |
| Whey protein | 3 | 45.23546 |  |  |
| Haemoglobin | 3 | 54.49084 |  |  |
| Gelatin | 3 |  | 64.22477 |  |
| BSA | 3 |  | 72.63484 |  |
| Casein | 3 |  |  | 100.00000 |
| Sig. |  | .061 | .095 | 1.000 |
| Means for groups in homogeneous subsets are displayed. | | | | |
| a. Uses Harmonic Mean Sample Size = 3.000. | | | | |

1. **Effect of Metal ions**
2. **Values of protease activity and relative activity of kinema protease in presence of metal ions**

| Metal Ions | Concentration  (mM) | PA1  (U/ml) | PA2  (U/ml) | PA3  (U/ml) | RA1  ( %) | RA2  (%) | RA3  (%) | Mean  RRA (%) | SD |
| --- | --- | --- | --- | --- | --- | --- | --- | --- | --- |
| Control | - | 0.375 | 0.376 | 0.376 | 100.000 | 100.000 | 100.000 | 100.000 | 0.000 |
| Na⁺ | 5 | 0.407 | 0.407 | 0.403 | 108.545 | 108.348 | 107.260 | 108.051 | 0.693 |
| Cu⁺^⁺^ | 5 | 0.302 | 0.311 | 0.322 | 80.545 | 82.759 | 85.662 | 82.989 | 2.566 |
| Fe⁺⁺⁺ | 5 | 0.234 | 0.245 | 0.229 | 62.364 | 65.154 | 60.980 | 62.833 | 2.126 |
| Hg⁺⁺ | 5 | 0.139 | 0.149 | 0.135 | 37.091 | 39.564 | 35.935 | 37.530 | 1.854 |
| Co⁺⁺ | 5 | 0.338 | 0.360 | 0.372 | 90.182 | 95.826 | 98.911 | 94.973 | 4.427 |
| Al⁺⁺⁺ | 5 | 0.177 | 0.136 | 0.163 | 47.091 | 36.298 | 43.376 | 42.255 | 5.483 |
| Ca⁺⁺ | 5 | 0.371 | 0.385 | 0.359 | 98.909 | 102.359 | 95.644 | 98.971 | 3.358 |
| K⁺ | 5 | 0.290 | 0.296 | 0.303 | 77.273 | 78.766 | 80.762 | 78.934 | 1.751 |
| Zn⁺⁺ | 5 | 0.365 | 0.387 | 0.376 | 97.455 | 103.085 | 100.181 | 100.240 | 2.816 |

| 1. **Tests of Normality** | | | | | | |
| --- | --- | --- | --- | --- | --- | --- |
|  | Kolmogorov-Smirnov^a^ | | | Shapiro-Wilk | | |
|  | Statistic | df | Sig. | Statistic | df | Sig. |
| Metalions | .104 | 30 | .200^*^ | .940 | 30 | .089 |
| *. This is a lower bound of the true significance. | | | | | | |
| a. Lilliefors Significance Correction | | | | | | |


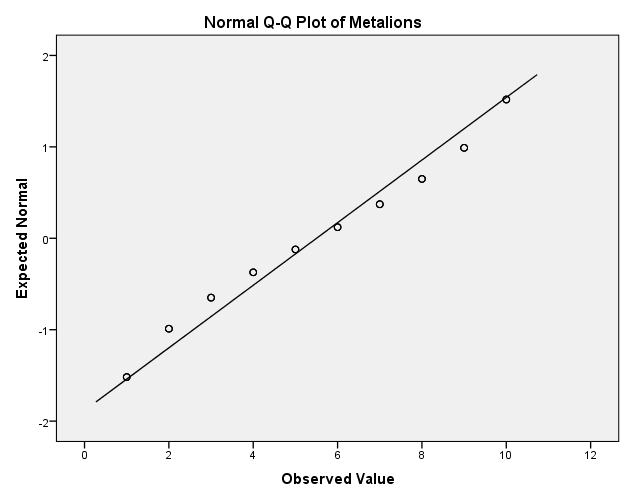

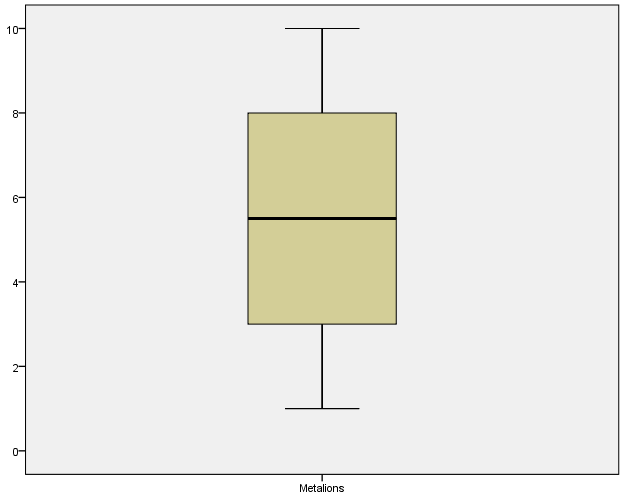


| 1. **Test of Homogeneity of Variances** | | | |
| --- | --- | --- | --- |
| RA | | | |
| Levene Statistic | df1 | df2 | Sig. |
| 1.941 | 9 | 20 | .104 |

| 1. **ANOVA** | | | | | |
| --- | --- | --- | --- | --- | --- |
| RA | | | | | |
|  | Sum of Squares | df | Mean Square | F | Sig. |
| Between Groups | 17127.567 | 9 | 1903.063 | 218.874 | .000 |
| Within Groups | 173.896 | 20 | 8.695 |  |  |
| Total | 17301.463 | 29 |  |  |  |

1. **Homogeneous subsets obtained from Post hoc test (TuKey HSD)**

| **RA** | | | | | | |
| --- | --- | --- | --- | --- | --- | --- |
| Tukey HSD | | | | | | |
| Metalions | N | Subset for alpha = 0.05 | | | | |
|  |  | 1 | 2 | 3 | 4 | 5 |
| Hg | 3 | 37.53000 |  |  |  |  |
| Al | 3 | 42.25500 |  |  |  |  |
| Fe | 3 |  | 62.83267 |  |  |  |
| K | 3 |  |  | 78.93367 |  |  |
| Cu | 3 |  |  | 82.98867 |  |  |
| Co | 3 |  |  |  | 94.97300 |  |
| Ca | 3 |  |  |  | 98.97067 |  |
| Control | 3 |  |  |  | 100.00000 | 100.00000 |
| Zn | 3 |  |  |  | 100.24033 | 100.24033 |
| Na | 3 |  |  |  |  | 108.05100 |
| Sig. |  | .631 | 1.000 | .792 | .494 | .074 |
| Means for groups in homogeneous subsets are displayed. | | | | | | |
| a. Uses Harmonic Mean Sample Size = 3.000. | | | | | | |

1. **Storage stability**
2. **Values of protease activity and relative activity of kinema protease stored at 4°C**

| Day | PA1 | PA2 | PA3 | RA1 | RA2 | RA3 | Relative Activity (%) | |
| --- | --- | --- | --- | --- | --- | --- | --- | --- |
| 0 | 1.070 | 1.000 | 1.053 | 100.000 | 100.000 | 100.000 | 100.000 | 0.000 |
| 2 | 0.588 | 0.633 | 0.604 | 54.972 | 63.258 | 57.326 | 58.519 | 4.270 |
| 4 | 0.446 | 0.609 | 0.532 | 41.626 | 60.901 | 50.477 | 51.002 | 9.648 |
| 6 | 0.423 | 0.425 | 0.424 | 39.521 | 42.496 | 40.237 | 40.751 | 1.553 |
| 8 | 0.361 | 0.362 | 0.355 | 33.722 | 36.222 | 33.685 | 34.543 | 1.454 |
| 10 | 0.364 | 0.282 | 0.393 | 34.014 | 28.215 | 37.274 | 33.167 | 4.588 |
| 12 | 0.243 | 0.276 | 0.255 | 22.708 | 27.626 | 24.202 | 24.845 | 2.521 |
| 14 | 0.191 | 0.224 | 0.228 | 17.881 | 22.392 | 21.666 | 20.646 | 2.422 |
| 16 | 0.188 | 0.261 | 0.228 | 17.557 | 26.135 | 21.673 | 21.788 | 4.290 |

1. **Values of protease activity and relative activity of kinema protease stored at -20°C**

| Day | PA1 | PA2 | PA3 | RA1 | RA2 | RA3 | Relative Activity (%) | |
| --- | --- | --- | --- | --- | --- | --- | --- | --- |
| 0 | 1.070 | 1.000 | 1.053 | 100.000 | 100.000 | 100.000 | 100.000 | 0.000 |
| 2 | 0.778 | 0.705 | 0.787 | 72.692 | 70.433 | 74.745 | 72.623 | 2.157 |
| 4 | 0.702 | 0.648 | 0.662 | 65.565 | 64.818 | 62.858 | 64.414 | 1.398 |
| 6 | 0.492 | 0.493 | 0.492 | 45.999 | 49.289 | 46.757 | 47.348 | 1.723 |
| 8 | 0.442 | 0.500 | 0.534 | 41.335 | 49.983 | 50.741 | 47.353 | 5.226 |
| 10 | 0.372 | 0.454 | 0.455 | 34.726 | 45.338 | 43.201 | 41.088 | 5.612 |
| 12 | 0.469 | 0.398 | 0.423 | 43.797 | 39.792 | 40.171 | 41.253 | 2.211 |
| 14 | 0.508 | 0.286 | 0.354 | 47.489 | 28.562 | 33.652 | 36.568 | 9.795 |
| 16 | 0.451 | 0.321 | 0.319 | 42.112 | 32.097 | 30.326 | 34.845 | 6.355 |
