# Supplementary material for: Partial purification and characterization of protease extracted from kinema
Source: Heliyon. 2024 Feb 27;10(5):e27173. doi: 10.1016/j.heliyon.2024.e27173 (PMC10923713; doi:10.1016/j.heliyon.2024.e27173)

**Supplementary Informations**

1. **Protease activity (Velocity of reaction) as a function of substrate concentration**

| Concentration  (mg/ml) | Velocity of Reaction ( R) µmole tyrosine release/ml/min | | |
| --- | --- | --- | --- |
|  | V1 | V2 | V3 |
| 30.0 | 0.924 | 0.930 | 0.927 |
| 20.0 | 0.886 | 0.934 | 0.960 |
| 10.0 | 0.969 | 0.956 | 0.962 |
| 5.0 | 0.992 | 0.856 | 0.924 |
| 2.5 | 0.764 | 0.703 | 0.782 |
| 1.0 | 0.570 | 0.626 | 0.498 |
| 0.5 | 0.361 | 0.340 | 0.350 |
| 0.25 | 0.190 | 0.221 | 0.206 |

1. R: command used to fit the data into Michaelis -Menten model

mm <- structure(list(S = c(30, 20, 10, 5, 2.5, 1,0.5,0.25,

30, 20, 10, 5, 2.5, 1, 0.5, 0.25,

30, 20, 10, 5, 2.5, 1, 0.5, 0.25, 0),

v = c(0.924, 0.886, 0.969, 0.992, 0.764, 0.570, 0.361, 0.190,

0.930, 0.934, 0.956, 0.856, 0.703, 0.626, 0.340, 0.221,

0.927, 0.960, 0.962, 0.924, 0.782, 0.498, 0.350, 0.206,0)),

.Names = c("S", "v"), class = "data.frame", row.names = c(NA, -25L))

#we used package drm to fit the model and package ggplot2 to draw the result. We load the necessary libraries.

library(drc) # for fitting Michaelis Menten model

library(ggplot2) # for drawing

#we fitted the data using function drm. We said that v depends on S, and the model that should be fitted is a two parameter Michaelis-Menten model (coded in functoin MM.2). After the model has been fitted, we predict the values in order to get a smooth fitted line.

model.drm <- drm (v ~ S, data = mm, fct = MM.2())

mml <- data.frame(S = seq(0, max(mm$S), length.out = 100))

mml$v <- predict(model.drm, newdata = mml)

#Using the below code, we can visualize the result

ggplot(mm, aes(x = S, y = v)) +

theme_bw() +

xlab("Concentration [mg/ml]") +

ylab("µmole tyrosine released/ml/min") +

ggtitle("Michaelis-Menten kinetics") +

geom_point(alpha = 0.5) +

geom_line(data = mml, aes(x = S, y = v), colour = "red")

#to save the result in a nifty pdf, use the below command

ggsave("mm.pdf", width = 6, height = 4)

#Using nls()We need to provide the starting values Vm and K.

model.nls <- nls(v ~ Vm * S/(K+S), data = mm,

start = list(K = max(mm$v)/2, Vm = max(mm$v)))

summary (model.drm)

summary (model.nls )

Parameters:

Estimate Std. Error t value Pr(>|t|)

K 0.82504 0.06973 11.83 2.93e-11 ***

Vm 1.00125 0.01802 55.56 < 2e-16 ***


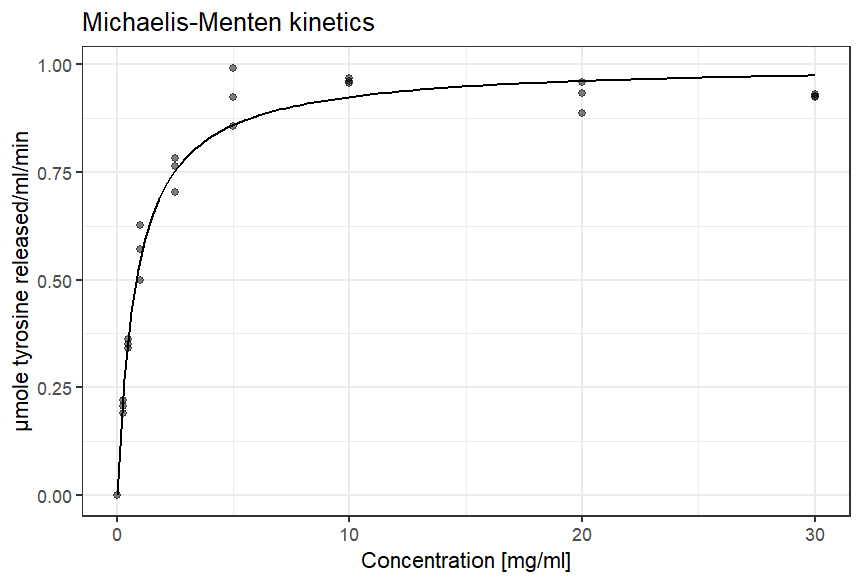


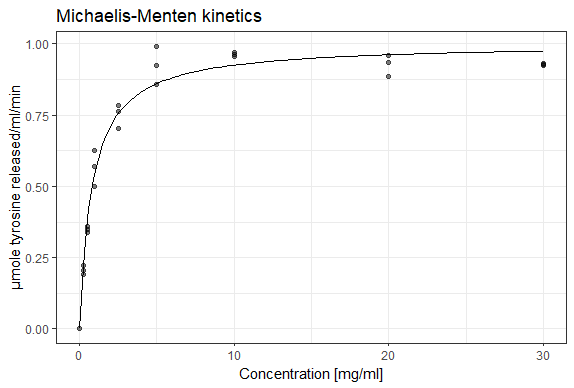

Supplement: Multimedia component 2 [file mmc2.docx]
