# Supplementary material for: Partial purification and characterization of protease extracted from kinema
Source: Heliyon. 2024 Feb 27;10(5):e27173. doi: 10.1016/j.heliyon.2024.e27173 (PMC10923713; doi:10.1016/j.heliyon.2024.e27173)
Supplement: Multimedia component 3 [file mmc3.doc]

Supplement _ Fig 9-A & B


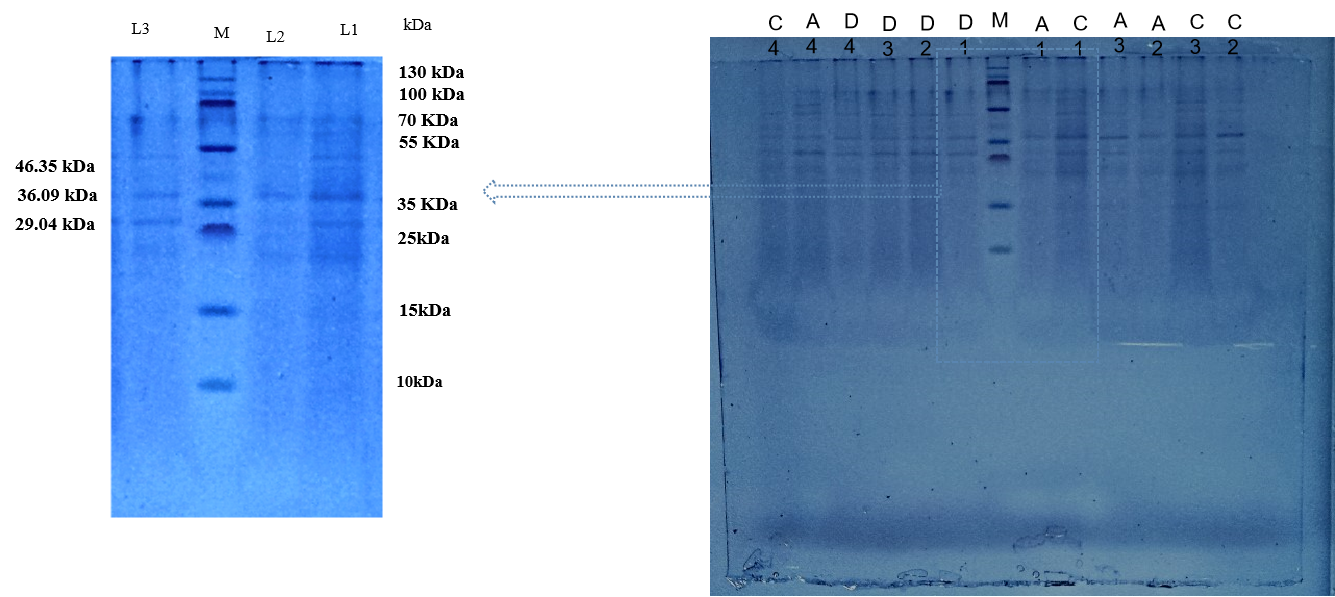


**Supplementary Fig. 9A**: Electrophoretic analysis of *kinema* protease extract using Tricine SDS-PAGE. Crude *kinema* extract, 30-70 % Ammonium sulfate precipitate (A), and dialysed *kinema* protease (D) obtained from four independent purifications (represented as 1, 2, 3 and 4 in figure) were loaded at same concentration of 10 µg to confirm the presence of protease. Almost similar patterns of protease distribution were observed among all the obtained dialysed samples. Hence the only the portion enclosed in the rectangular box was taken to express the results.


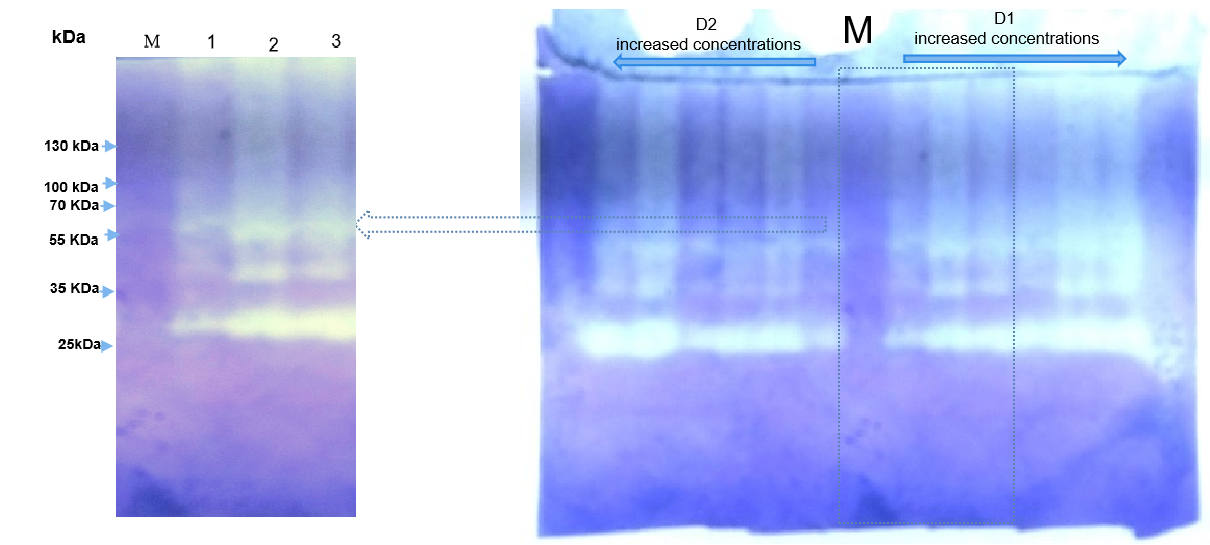


**Supplementary Fig. 9B** Gelatin zymogram of dilalysed kinema extract. After observing similar patterns protein distribution in dialysed samples from SDS –PAGE, The dialysed samples D1 and D2 were loaded at various increasing concentrations (5, 7, 10, 12, 15 and 20 µg) to perform the gelatin zymography. Both the samples showed almost increased in white band intensity with increased concentrations representing three active bands and in higher concentration (at 15 and 20µg) the representing lane columns were observed highly whitened. Hence only the portion enclosed in the rectangular box of sample D1 at lower concentrations (5, 7 and 10 µg) was taken to express the results.
